# HuR‐positive stress granules: Potential targets for age‐related osteoporosis

**DOI:** 10.1111/acel.14053

**Published:** 2024-02-20

**Authors:** Ying Huai, Xue Wang, Wenjing Mao, Xuehao Wang, Yipu Zhao, Xiaohua Chu, Qian Huang, Kang Ru, Ling Zhang, Yu Li, Zhihao Chen, Airong Qian

**Affiliations:** ^1^ Lab for Bone Metabolism, Xi'an Key Laboratory of Special Medicine and Health Engineering Northwestern Polytechnical University Xi'an China; ^2^ Key Lab for Space Biosciences and Biotechnology, Research Center for Special Medicine and Health Systems Engineering Northwestern Polytechnical University Xi'an China; ^3^ NPU‐UAB Joint Laboratory for Bone Metabolism, School of Life Sciences Northwestern Polytechnical University Xi'an China; ^4^ Department of Orthopedics Tangdu Hospital, Air Force Military Medical University Xi'an China

**Keywords:** age‐related osteoporosis, apigenin, HuR, osteogenesis, stress granules

## Abstract

Aging impairs osteoblast function and bone turnover, resulting in age‐related bone degeneration. Stress granules (SGs) are membrane‐less organelles that assemble in response to stress via the recruitment of RNA‐binding proteins (RBPs), and have emerged as a novel mechanism in age‐related diseases. Here, we identified HuR as a bone‐related RBP that aggregated into SGs and facilitated osteogenesis during aging. HuR‐positive SG formation increased during osteoblast differentiation, and HuR overexpression mitigated the reduction in SG formation observed in senescent osteoblasts. Moreover, HuR positively regulated the mRNA stability and expression of its target β‐catenin by binding and recruiting β‐catenin into SGs. As a potential therapeutic target, HuR activator apigenin (API) enhanced its expression and thus aided osteoblasts differentiation. API treatment increased HuR nuclear export, enhanced the recruitment of β‐catenin into HuR‐positive SGs, facilitated β‐catenin nuclear translocation, and contributed osteogenesis. Our findings highlight the roles of HuR and its SGs in promoting osteogenesis during skeletal aging and lay the groundwork for novel therapeutic strategies against age‐related skeletal disorders.

## Graphical abstract

This study has contributed to our understanding of the role of HuR and SGs in bone homeostasis. By demonstrating the positive regulation of HuR on osteogenesis and the formation of HuR‐positive SGs in osteoblasts, this study highlights HuR and its SGs as potential therapeutic targets for age‐related bone loss.

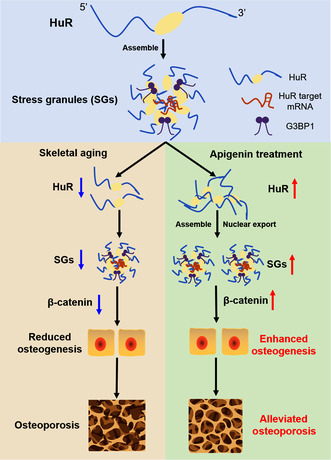

**Abbreviations**
ActD: actinomycinD
ADME: absorption, distribution, metabolism and excretion
ALPL: alkaline phosphatase
α‐MEM: alpha‐minimum essential medium
API: apigenin
ARS: alizarin redS
BMD: bone mineral density
BMSCs: bone marrow‐derived mesenchymal stem cells
BV/TV: bone volume/tissue volume
CMC‐Na: sodium carboxymethyl cellulose
Co‐IP: co‐immunoprecipitation
COL1A1: collagen type I alpha 1
CSF‐1R: colony‐stimulating factor 1 receptor
DAPI: 4’,6‐diamidino‐2‐phenylindole
DMSO: dimethylsulfoxide
FBS: fetal bovine serum
G3BP1: GTPase activating protein (SH3 domain) binding protein 1
GAPDH: glyceraldehyde‐3‐phosphate dehydrogenase
H&E: hematoxylin‐eosin
HPLC: high purity liquid chromate
HuR: human antigen R
IDRs: intrinsically disordered regions
IgG: immunoglobulin G
IHC: immunohistochemical
LLPS: liquid‐liquid phase separation
Micro‐CT: micro‐computed tomography
MLOs: membrane‐less organelles
NHS: N‐hydroxysuccinimide
OCN: osteocalcin
OVX: ovariectomized
PLDs: prion‐like domains
QKI‐5: Quakingi‐5
RANK: receptor activator of nuclear factor kappa B
RBDs: RNA‐binding domains
RBPs: RNA binding proteins
RUNX2: runt‐related transcription factor 2
SA: sodium arsenite
SGs: stress granules
SP7: Sp7 transcription factor
SPR: surface plasmon resonance
Tb.N: trabecular number
Tb.Sp: trabecular spacing
Tb.Th: trabecular thickness
TIA1: T‐cell intracellular antigen 1

## 1 INTRODUCTION

The skeletal system undergoes significant changes during aging, including alterations in bone structure and osteoblastic dysfunction, which contribute to age‐related bone loss (Lorentzon et al., 2022). The mechanisms underlying skeletal aging, however, remain poorly understood, and current treatments for age‐related osteoporosis were limited. Stress granules (SG), prototypical cytoplasmic membrane‐less organelles (MLOs) that dynamically assemble in response to various stresses, is an emerging area of interest (Mahboubi & Stochaj, 2017; Protter & Parker, 2016). Aberrant SG formation has been proposed as a novel mechanism underlying aging and its associated pathologies (Cao et al., 2020; Marcelo et al., 2021). RNA‐binding proteins (RBPs) have been identified as the central scaffold proteins driving SG assembly. One such RBP is human antigen R (HuR), which aggregates into SGs and is a well‐established mRNA stabilizer (Corley et al., 2020; Gebauer et al., 2021; Ma et al., 1996) possessing RNA‐binding domains (RBDs), intrinsically disordered regions (IDRs), and prion‐like domains (PLDs).

The roles of HuR in various bone diseases are gradually being unveiled. For instance, HuR has been shown to play a significant role in osteosarcoma cell migration and epithelial–mesenchymal transition (Liu et al., 2021). Lee et al. demonstrated that knockdown of HuR could restrain the bone metastasis and osteolysis in mice with metastatic breast cancer (Lee et al., 2017). Another study revealed that proper exercise modulates HuR expression in aging skeletal muscle, thereby stabilizing various mRNAs and delaying skeletal muscle atrophy (Cammas et al., 2014; Wang et al., 2019). Moreover, our recent study identified HuR as an essential osteoporosis‐related RBP (Huai et al., 2022). However, the exact roles of HuR and its SGs in regulating bone metabolism remains an enigma, prompting further investigation into the function of HuR in osteoporotic bone regeneration.

In this study, we aimed to explore the potential roles of HuR and SGs in bone metabolism and regeneration. We observed positive correlations between HuR levels and bone mineral density (BMD), bone formation markers, and SGs formation markers, while noting a negative correlation with age in bone specimens from postmenopausal women. Our investigations revealed that HuR is a novel bone‐related RBP that positively correlates with osteoblast differentiation. We observed a decline in both HuR expression and formation of HuR‐positive SGs in primary osteoblasts from aging mice, which was rescued by overexpression of HuR. Inhibition of HuR‐positive SG formation resulted in a decline of osteoblastic differentiation, suggesting the crucial roles of HuR and SGs in osteogenesis.

To further explore the potential therapeutic applications of HuR and SGs in bone regeneration, we identified apigenin (API) as a HuR activator that enhanced HuR expression and bone formation both in vivo and in vitro. We proposed a novel mechanism whereby cytoplasmic accumulation of HuR induced by API triggers SG formation, while also recruiting β‐catenin into SGs, ultimately promoting β‐catenin levels and nuclear translocation to improve the anabolic action of bones during aging. Our findings highlight the crucial roles of HuR and SGs in osteogenesis and establish a novel therapeutic strategy for age‐related osteoporosis.

Overall, our study provides novel insights into the roles of HuR and SGs in osteogenesis, highlighting their potential therapeutic applications in treating age‐related osteoporosis. These findings suggest that the manipulation of HuR and SGs formation may offer a promising strategy to enhance bone formation and regeneration in aging individuals.

## 2 MATERIALS AND METHODS

### 2.1 Dataset collection and data pre‐processing

The transcriptome dataset (E‐MEXP‐1618) was retrieved from the online functional genomics database ArrayExpress. We used the original grouped data of the published work for the subsequent analysis. The raw data were processed and normalized using the “affy” package in R program.

### 2.2 Molecular docking

Molecular docking is one of the common approaches to illuminate the binding modes between the small molecules and their targets. In this section, we performed molecular docking simulations using program AutoDock Vina (v.1.2.0). The x‐ray crystal structures of the HuR was extracted from RCSB Protein Data Bank (www.rcsb.org). The SDF files of natural products were downloaded from PubChem‐compound database. Following pre‐processing to delete crystallographically observed water molecules, hydrogen atoms and charges were added to HuR protein and compound API using AutoDock tools software. Next, grid boxes were defined for API (120 × 120 × 120 Å) that covered the whole receptor structure to perform a blind molecular docking simulation. The docking parameters were set as the default. Finally, the docking results were analyzed and visualized by PyMOL software.

### 2.3 Surface plasmon resonance (SPR) analysis of HuR and API

To determine the binding of API to HuR protein, we used a BIAcore T200 instrument (GE Healthcare) with a CM5 sensor chip (GE Healthcare) to conduct the SPR analysis. The activation, deactivation, preparation of the coupled flow cell, and ligand‐binding assay were performed as previously reported (Li et al., 2017). Briefly, HuR purified protein was immobilized in the parallel‐flow channels of a BIAcore™ CM5 sensor chip using an amine coupling kit (GE Healthcare) at a flow rate of 10 μL/min in 10 mM sodium acetate buffer (pH 4.5). The sensor surface was activated for 7 min. A mixture of 50 mM N‐hydroxysuccinimide (NHS) and 200 mM 1‐ethyl‐3‐(3‐dimethylaminopropyl) carbodiimide (EDC) was injected. Thereafter, 20 μg/mL of HuR purified protein was injected to reach the target level of 8420.8 RU, after which the surface was blocked with 1 M ethanolamine (pH 8.5). To check the binding of HuR to API, serial dilutions of API with different concentrations were injected into the flow system. All binding analysis was performed in PBSP with 0.05% (v/v) Tween‐20 and 1% Dimethyl sulfoxide (DMSO) at pH 7.4 and 25°C. Prior to analysis, double reference subtractions and solvent corrections were made to eliminate bulk refractive index changes, injection noise, and data drift from the analysis. The binding affinity was determined by fitting the data to a Langmuir 1:1 binding model using the Biacore Evaluation software (GE Healthcare).

### 2.4 Cell staining

#### 2.4.1 Alkaline phosphatase (ALP) staining

ALP staining was performed with a BCIP/NBT Alkaline Phosphatase Color Development Kit (Beyotime, China) according to the manufacturer's instructions. Briefly, cells were carefully rinsed with PBS three times and fixed with 4% paraformaldehyde for 20 min. The fixed cells were washed with PBS three times again. Then, BCIP/NBT liquid substrate was added to each cell well to staining for 20–30 min. Finally, the cells were washed with ddH_2_O after the color turned blue/purple. The stained cell plates were imaged by a scanner (Canon, Japan).

#### 2.4.2 Alizarin red S (ARS) staining

Cells were carefully rinsed with PBS three times and fixed with 4% paraformaldehyde for 20 min. The fixed cells were washed with PBS three times again and stained with 0.5% alizarin red S (Sigma, USA) solution (pH 4.2) for 15–30 min at room temperature. After washing with ddH_2_O four times, the cell plates with mineralized nodules were imaged by a scanner (Canon, Japan).

### 2.5 Immunocytochemistry staining

For immunocytochemistry staining, cells were first fixed with either 4% PFA for 20 min at room temperature followed by permeabilization with 0.5% Triton X‐100 for 10 min. Next, samples were blocked with 2% BSA diluted in 0.1% Triton X‐100 for 10 min and sequentially incubated with primary rabbit anti‐β‐catenin (1:50) antibodies overnight at 4°C. After washing with 0.1% Triton X‐100, cells were incubated with PE‐conjugated goat anti‐rabbit IgG secondary antibodies (1:200, Hangzhou Hua A Biotechnology, Hangzhou, China) for 120 min at room temperature. Finally, 4′,6‐diamidino‐2‐phenylindole (DAPI, 1 μg/mL) was used to counterstain cell nuclei for 3 min at room temperature. Each sample was examined with an inverted fluorescence microscope (Leica DMIL, Wetzlar, Germany).

### 2.6 Determination of mRNA stability

Actinomycin D (ActD) chase experiment was performed for β‐catenin or *Hur* mRNA stability assay within MC3T3‐E1 cells. Total RNA was isolated at various time points following addition of ActD (Aladdin, A113142, China) with a final concentration of 5 μg/mL. Real‐time PCR was used to quantify the mRNA levels. *Gapdh* was used as internal controls for normalization.

### 2.7 Co‐immunoprecipitation (Co‐IP)

A co‐immunoprecipitation kit with Protein A + G Magnetic Beads (Beyotime, CN) was used to validate the interaction between HuR protein and β‐catenin as well as G3BP1. In brief, magnetic beads were coated with 5 μg of antibodies, including anti‐HuR (Proteintech, CN) and anti‐immunoglobulin G (IgG) (Beyotime, CN), for 30 min at room temperature. The cell lysate from 2 × 10^7^ cells was incubated with antibody‐coated magnetic beads overnight at 4°C to form beads antigen–antibody complexes. After washing away nonspecific proteins, the immunoprecipitated complexes are eluted and subjected to western blotting analysis to study protein–protein interactions.

### 2.8 Micro‐computed tomography (Micro‐CT) analysis

To visualize the bone microarchitecture of the aging mice, femur samples fixed in 4% paraformaldehyde were analyzed by micro‐CT (General Electric, WI). The scanners were set at an isotropic resolution of 8 μm (energy: 80 kVp/80 μA; angle of increment: 0.5°; exposure time: 3000 ms/frame; and scanning time: 120 min) for the fixed bone samples. The built‐in software was acquired for data reconstruction. After scanning, a region of interest was selected 1 mm above the distal growth plate. Finally, the following parameters of the trabecular bone were calculated by the MicroView software: bone mineral density (BMD; g/cm^3^), bone volume/tissue volume (BV/TV; %), trabecular thickness (Tb.Th; mm), trabecular number (Tb.N; mm), and trabecular spacing (Tb.Sp; mm).

### 2.9 Bone mechanical properties

The biomechanical properties of the tibia bone were examined by the three‐point bending mechanical test system (UniVert, Canada), as shown in Figure 7f. The dissected tibias were wrapped with gauze dipped in saline for biomechanical testing. The tibias were kept wet and placed on two lower support points with a span distance of 10 mm. The UniVert mechanical test system was used to apply bending moments to the front and rear surfaces at a constant displacement rate of 0.6 mm/min up to a three‐point bending displacement of 0.5 mm. The mechanical data were acquired and used to determine the parameters of mechanical properties including maximum load, stiffness, and elastic modulus.

### 2.10 Bone histochemistry

#### 2.10.1 Double calcein labeling

To assess the bone formation rate in the mice, calcein (Sigma, USA) was injected (10 mg/kg body weight) twice before euthanasia. Subsequently, femur samples were isolated and processed into undecalcified plastic sections. These sections were then examined with a fluorescence microscope (Nikon, Japan).

#### 2.10.2 Bone histological decalcified sections staining

For the histological analysis of bone samples, the femurs were fixed with 4% PFA for 2 days, decalcified with EDTA for two weeks, and then embedded in paraffin. Serial tissue sections, measuring 4 μm in thickness, were prepared for staining purposes. Hematoxylin and eosin (HE) staining or immunohistochemical (IHC) staining following the manufacturer's instructions (Servicebio, China) were performed as previous described. The stained sections were scanned by a Digital Whole Slide Scanner (Leica, Germany).

### 2.11 Statistical analysis

For data consisting of three or more groups were analyzed via one‐way ANOVA. Significance between two groups was determined using Student's *t* test. Statistical analyses of the data were performed using the GraphPad Prism 8 software (GraphPad Software, La Jolla, CA). All data were reported as the mean ± standard deviation (SD), and the significance level was set to a 95% confidence interval (*p < 0.05, **p < 0.01, ***p < 0.001).

## 3 RESULTS

### 3.1 HuR levels and HuR‐positive SGs were positively associated with bone formation

**FIGURE 1:**
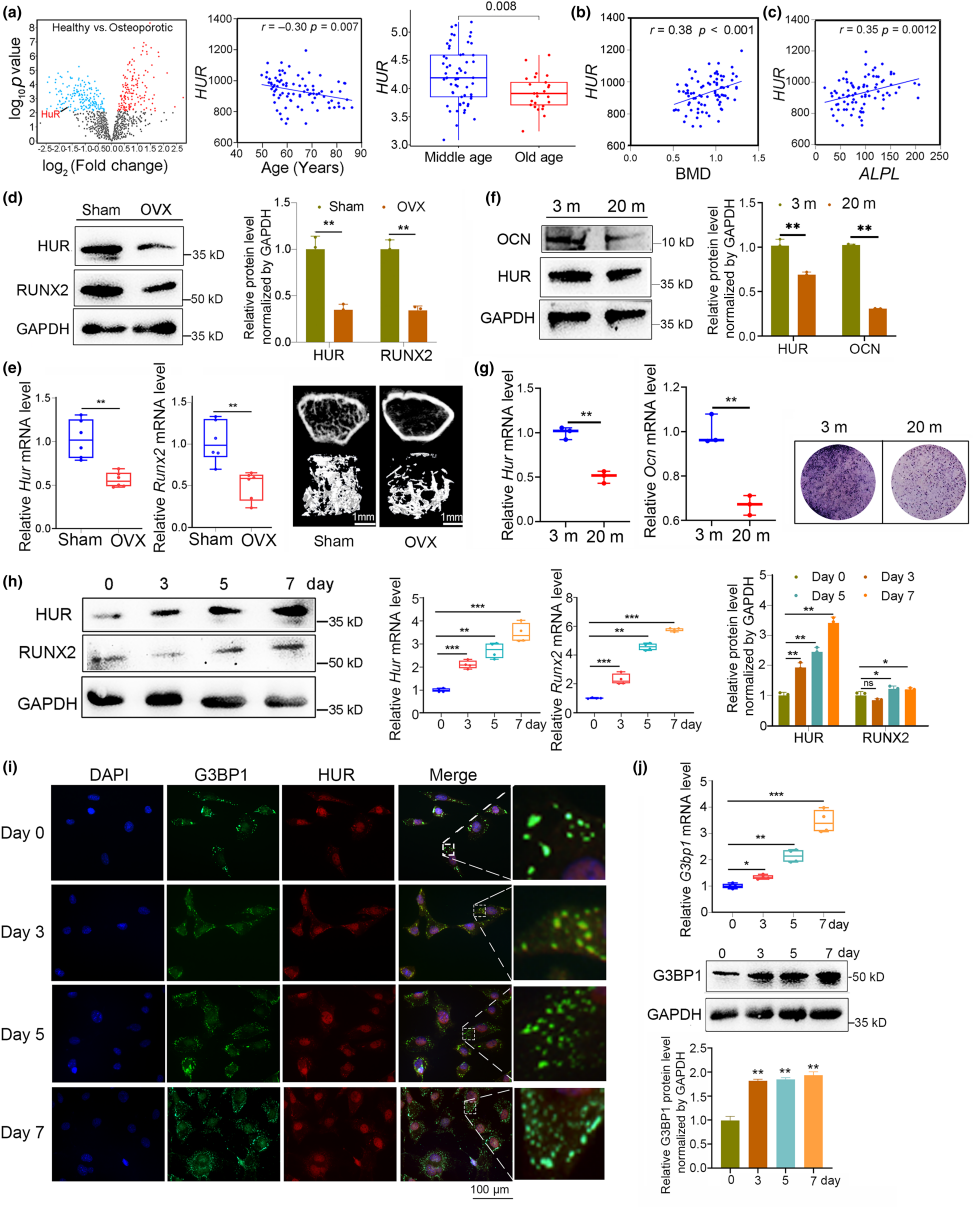
HuR levels and HuR‐positive SGs were positively associated with bone formation. (a) The volcano plot analysis of differentially expressed genes and HuR in dataset of E‐MEXP‐1618 (Left). The linear regression analysis of HuR levels and age association in bone specimens of healthy patients (T‐score ≥−1) or postmenopausal osteoporotic patients (T‐score ≤−2.5) in dataset of E‐MEXP‐1618 (middle) as well as the HuR expression level in middle‐aged patients and elderly patients in dataset of E‐MEXP‐1618 (Right). (b) The linear regression analysis of HuR level and BMD in bone specimens of healthy patients or postmenopausal osteoporotic patients in dataset of E‐MEXP‐1618. (c) The linear regression analysis of HuR level and bone formation biomarker ALPL association in bone specimens of healthy patients or postmenopausal osteoporotic patients in dataset of E‐MEXP‐1618. (d, e) Western blot and real‐time PCR analyses of HUR and Runx2 protein levels and mRNA levels in bone specimens from OVX‐induced osteoporosis mice (Sham: *n* = 6; OVX: *n* = 6). Representative images showing trabecular microarchitecture, scale bar: 1 mm. (f, g) Western blot and real‐time PCR analyses of HUR and osteocalcin (OCN) protein levels and mRNA levels in the primary osteoblasts obtained from 3‐month and 20‐month mice (3 m: *n* = 3; 20 m: *n* = 3). Representative images showing ALP activities of the primary osteoblasts. (h) Western blot and real‐time PCR analyses of HUR and RUNX2 protein levels and mRNA levels during osteoblasts differentiation (0–7 days) of MC3T3‐E1 cells. *n* = 3. (i) Cell immunofluorescence analysis of HuR‐positive SGs formation during osteogenesis (0–7 day). G3BP1: green fluorescence; HuR: red fluorescence. *n* = 3. Scale bar: 100 μm. (j) Real‐time PCR and western blot analyses of SGs biomarker gene (G3BP1) mRNA levels (upper) and protein levels (below) during osteogenesis (0–7 day). *n* = 3. Glyceraldehyde‐3‐phosphate dehydrogenase (GAPDH) was used as the internal control for mRNA and protein. Data are represented as mean ± SD. Statistical differences among three groups were analyzed via one‐way ANOVA and significances were determined using Student's *t* test between two groups. *p < 0.05 was considered significant in all cases (**p < 0.01, ***p < 0.001).

In our previous study, we observed a decline in HuR levels in bone specimens obtained from aged osteoporotic patients and mice (Huai et al., 2022). Consistently, we found that HuR expression decreased with increasing age of patients (*r* = −0.31, *p* = 0.007) (Figure 1a). Furthermore, HuR exhibited positive correlations with bone mineral density (BMD) (*r* = 0.38, *p* < 0.001) and T‐scores (*r* = 0.40, *p* < 0.001) in bone specimens from postmenopausal women (Figure 1b and Figure S1A). Additionally, we identified positive correlations between HuR and osteogenic markers, including alkaline phosphatase (*ALPL*) (*r* = 0.35, *p* = 0.0012), Collagen type I Alpha 1 (*COL1Α1)* (*r* = 0.41, *p* < 0.001), Runt related transcription factor 2 (*RUNX2*) (*r* = 0.33, *p* = 0.002), and SP7 transcription factors (*SP7*) (*r* = 0.22, *p* = 0.04) (Figure 1c and Figure S1B). Subsequently, we verified a decline in HuR expression levels in bone specimens (Figure 1d) obtained from ovariectomized (OVX) mice, which exhibited deteriorative bone microarchitecture and decreased bone formation (Figure 1e). Concurrently, we observed a decrease in HuR expression levels in primary osteoblast cells derived from 20‐month‐old mice compared to those from 3‐month‐old mice (Figure 1f,g). This finding further supported the association between HuR levels and aging in the context of osteoblast function. To further investigate the roles of HuR in osteoblast differentiation, we examined the levels of HuR at different time points (0, 3, 5, and 7 days) during the process of osteogenic differentiation. We observed an upward trend in both transcriptional and translational levels of HuR and osteogenic markers (ALP and RUNX2) during MC3T3‐E1 cell osteogenesis (Figure 1h and Figure S1C). These findings suggest a positive association between HuR and bone formation as well as osteogenic differentiation, while indicating a negative correlation with advancing age.

The unique sequence and structural characteristics of HuR enable its translocation from the nucleus to the cytoplasm (Fan & Steitz, 1998), allowing for regulatory functions on cell cycle progression and mRNA fate (Figure S1D). Our prediction that the HuR protein harbors IDRs that play a pivotal role in SG assembly, along with its function for assembling and organizing SGs by recruiting its target mRNAs/proteins (Figure S1D,E) prompted us to investigate the regulatory roles of HuR in SG formation in osteoblasts. We initially noted a significant positive correlation between levels of HuR and G3BP1 (GTPase activating protein (SH3 domain) binding protein 1) as well as TIA1 (T‐cell intracellular antigen 1) (widely recognized biomarkers for SGs) in bone specimens from postmenopausal women (Figure S1F). Additionally, we observed a positive correlation between the expression of osteogenic markers (Runx2 and Col1α1) and the level of G3BP1 in bone specimens from postmenopausal women (Figure S1G). Conversely, the SGs marker (G3BP1 and TIA1) showed a positive correlation with the age of postmenopausal women. Additionally, osteogenic markers (Runx2 and Col1α1) expression exhibited a negative correlation with G3BP1 level in bone specimens from postmenopausal women (Figure S1G), while SGs markers demonstrated a positive association with the age of postmenopausal women (Figure S1H). Finally, we examined the presence of HuR‐positive SGs in MC3T3‐E1 cells following a 30‐min exposure to 500 mM sodium arsenite (SA), a well‐established inducer of SGs formation. Immunofluorescence staining demonstrated the co‐localization of HuR (red fluorescence) and G3BP1 (green fluorescence), as well as co‐localization of HuR (green fluorescence) and TIA1 (red fluorescence), indicating the presence of HuR within SGs in osteoblasts (Figure S1I). As HuR levels are positively correlated with osteogenic differentiation, it is worth investigating the formation of HuR‐positive SGs during this process. As expected, we observed an increasing trend in the formation of HuR‐positive SGs (red fluorescence) during osteoblast differentiation (0–7 days), indicating a positive association between the formation of these SGs and osteogenesis (Figure 1i). Additionally, there was a gradual increase in G3BP1 expression (both mRNA and protein levels) during osteogenesis (Figure 1j). In summary, these findings indicate that HuR aggregates into SGs in osteoblasts and that HuR‐positive SGs display a positive correlation with osteogenesis.

### 3.2 HuR positively regulated SGs formation and thus osteogenesis

**FIGURE 2:**
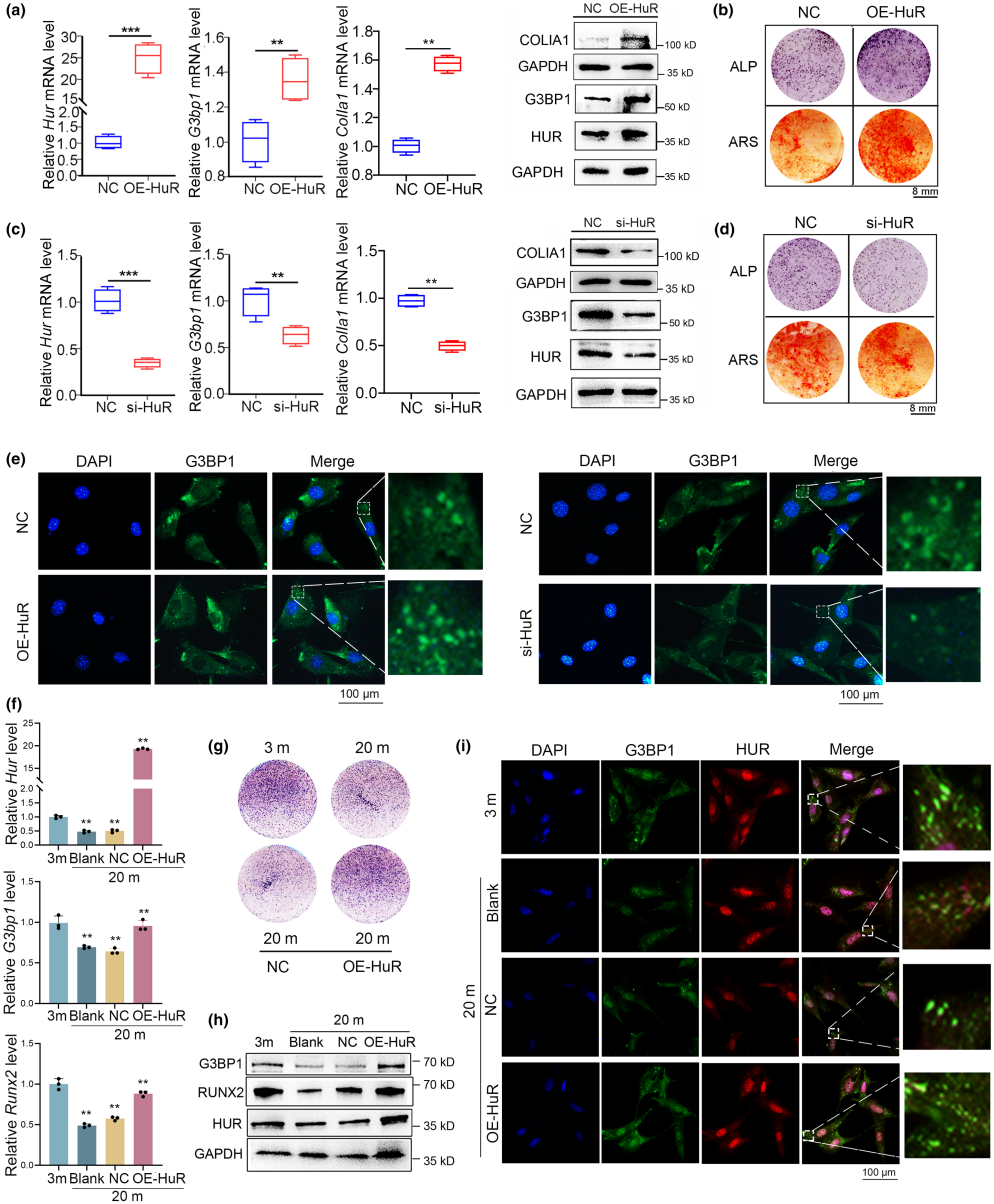
HuR positively regulated SGs formation and thus osteogenesis. (a, c) Real‐time PCR and western blot analyses of HUR, G3BP1, and CoLIA1 mRNA levels and protein levels in MC3T3‐E1 cells treating with HuR‐specific plasmid (OE‐HuR), HuR‐specific siRNA or NC for 48 h *n* = 3. (b, d) Representative images of ALP staining and Alizarin Red S (ARS) staining (10 d) in MC3T3‐E1 cells treating with OE‐HuR, HuR‐specific siRNA, or NC for 48 h *n* = 3. (e) Cell immunofluorescence analysis of G3BP1‐positive SGs formation in MC3T3‐E1 cells treating with HuR‐specific plasmid (OE‐HuR), HuR‐specific siRNA, or NC for 48 h. G3BP1: green fluorescence. *n* = 3. Scale bar: 100 μm. (f) Real‐time PCR analysis of *Hur*, *G3bp1*, and *Runx2* in 3‐month and 20‐month mouse primary osteoblasts as well as in 20‐month mouse primary osteoblasts transfected with HuR‐specific plasmids. *n* = 3. (g) Representative ALP staining images of 3‐month and 20‐month mouse primary osteoblasts as well as 20‐month mouse primary osteoblasts transfected with HuR‐specific plasmids. *n* = 3. (h) Western blot analysis of HUR, G3BP1, and RUNX2 in 3‐month and 20‐month mouse primary osteoblasts as well as in 20‐month mouse primary osteoblasts transfected with HuR‐specific plasmids. *n* = 3. (i) Cell immunofluorescence analysis of HuR‐positive SGs formation in 3‐month and 20‐month mouse primary osteoblasts as well as in 20‐month mouse primary osteoblasts transfected with HuR‐specific plasmids. G3BP1: green fluorescence; HuR: red fluorescence. *n* = 3. Scale bar: 100 μm. GAPDH was used as the internal control for mRNA and protein. Data are represented as mean ± SD. Statistical differences among three groups were analyzed via one‐way ANOVA and significances were determined using Student's *t* test between two groups. *p < 0.05 was considered significant in all cases (**p < 0.01, ***p < 0.001).

To further explore the impact of HuR on osteogenesis and SG formation, we modulated HuR levels by HuR‐specific plasmid or siRNA. Firstly, we found that HuR levels positively regulated the osteoblast differentiation, including the expression of osteogenic biomarkers (Figure 2a,c), ALP activity as well as mineralized matrix formation in osteoblasts (Figure 2b,d). Moreover, overexpression of HuR led to elevated levels of the SG formation marker G3BP1 (both at the transcriptional and translational levels) (Figure 2a) as well as an increase in the formation of G3BP1‐positive SGs (indicated by green fluorescence) (Figure 2e (Left)). Conversely, knockdown of HuR inhibited the level of SGs marker (G3BP1) and formation of G3BP1‐positive SGs (green fluorescence) in osteoblasts (Figure 2c,e (Right)). Therefore, we conclude that HuR level has positive regulatory effects on SGs formation and osteogenesis in vitro in osteoblasts.

We further investigated the potential impact of SG formation on osteogenesis by treating osteoblasts with menadione (a HuR SGs inhibitor). We found that menadione treatment significantly suppressed the cytoplasmic aggregation of HuR and the formation of HuR‐positive SGs in osteoblasts (Figure S2A). Notably, treatment with menadione resulted in significant reductions of HuR and G3BP1 expressions, as well as the decreased expressions of osteogenic markers (*Runx2*, *Sp7*, and *ColIα1*) and ALP activity in osteoblasts (Figure S2B,C). The above findings suggest that the reduction in HuR expression and disruption of SGs formation can lead to a significant impairment of osteogenesis.

Notably, we found that enhancing HuR expression could ameliorate the impaired osteogenesis in osteoblasts derived from 20‐month‐old mice, showing as the improved osteogenic marker levels and ALP activities (as depicted in Figure 2f (Upper and Middle) and Figure 2g,h). Moreover, high levels of HuR also alleviated the transcriptional and translational decreases of G3BP1 expression in primary osteoblasts from 20‐month‐old mice (Figure 2f (Bottom) and Figure 2h). Importantly, we also observed a decrease in the formation of HuR‐positive SGs with red fluorescence in primary osteoblasts from 20‐month‐old mice compared to those from 3‐month‐old mice. While the overexpression of HuR significantly promoted the HuR‐positive SGs formation in primary osteoblasts from 20‐month‐old mice (Figure 2i). These observations demonstrate that increased HuR levels effectively alleviate the reduction in SG formation and osteogenesis, implying that targeting HuR and its SGs could potentially revolutionize our approach to treating age‐related osteoporosis.

In summary, our results indicate that HuR levels have positive regulatory effects on SG formation and osteogenesis in vitro. Furthermore, the reduction of HuR expression and disruption of SG formation can lead to a significant impairment of osteogenesis. Our findings also suggest that HuR and SGs are potential therapeutic targets for age‐related osteoporosis.

### 3.3 HuR recruits and co‐localizes with β‐catenin in HuR‐positive SGs

**FIGURE 3:**
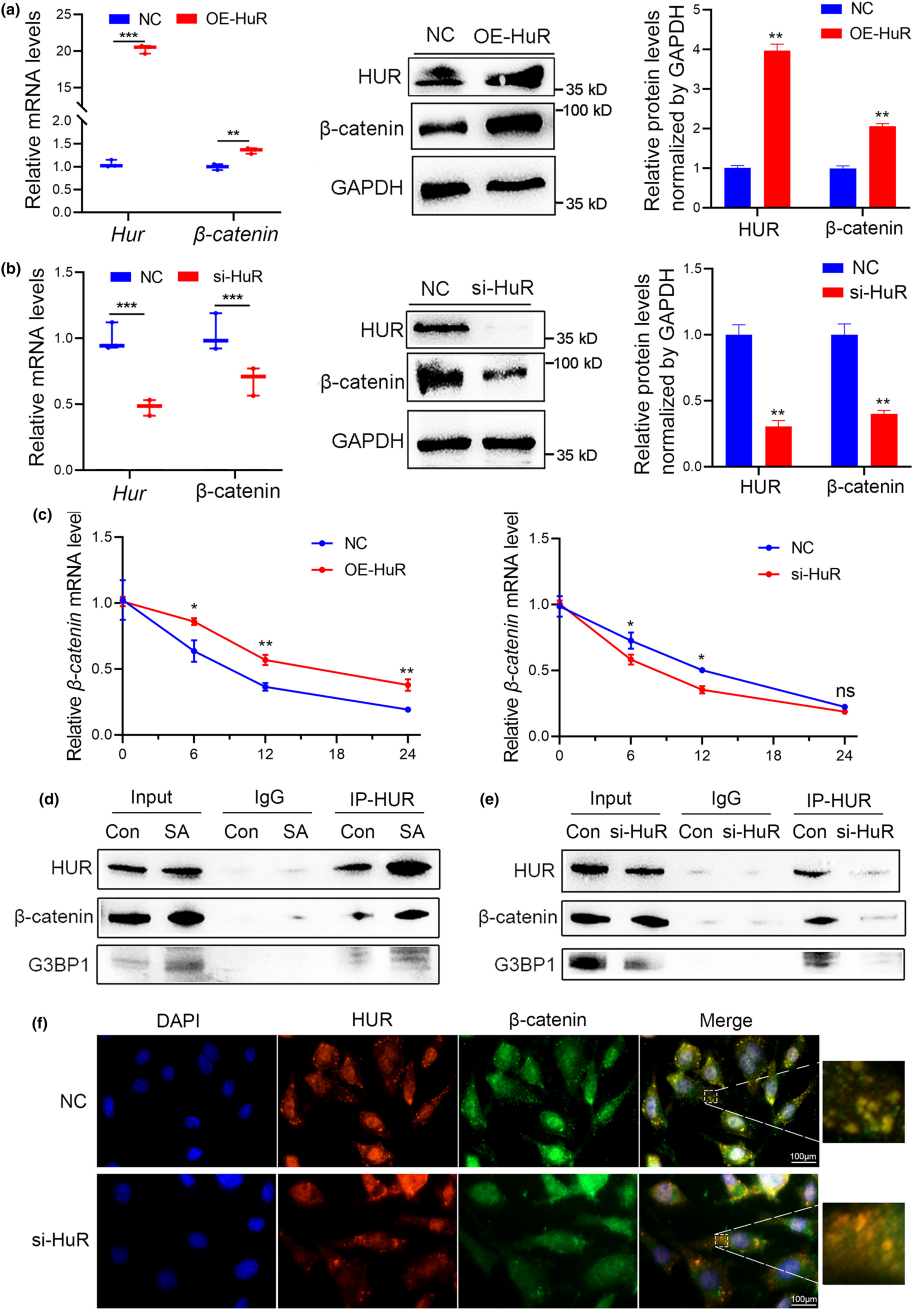
HuR recruits and co‐localizes with β‐catenin in HuR‐positive SGs. (a, b) Real‐time PCR and western blot analyses of HUR and β‐catenin levels in MC3T3‐E1 cells treating with HuR‐specific plasmid (OE‐HuR), HuR‐specific siRNA (si‐HuR) or NC for 48 h (left). *n* = 3. (c) The mRNA stability of β‐catenin in MC3T3‐E1 cells transfected with HUR‐specific plasmid (left) or siRNA (right) for 48 h, followed by exposure to ActD for the indicated periods of time (0, 6, 12, and 24 h). *n* = 3. (d) Co‐immunoprecipitation assay of the specific interaction between HuR and β‐catenin, as well as G3BP1 in MC3T3‐E1 cells following 30 min of exposure to 500 μM SA. *n* = 3. (e) Co‐immunoprecipitation assay of the specific interaction between HuR and β‐catenin, as well as G3BP1 in HuR‐knock down MC3T3‐E1 cells. *n* = 3. (f) Cell immunofluorescence analysis of the co‐localization of β‐catenin and HuR in HuR‐positive SGs in HuR‐knockdown MC3T3‐E1 cells. HuR: red fluorescence; β‐catenin: green fluorescence. *n* = 3. Scale bar: 100 μm. GAPDH was used as the internal control for mRNA and protein. Data are represented as mean ± SD. Statistical differences among three groups were analyzed via one‐way ANOVA and significances were determined using Student's *t* test between two groups. *p < 0.05 was considered significant in all cases (**p < 0.01, ***p < 0.001).

As a mRNA stabilizer, HuR forms ribonucleoprotein complexes with discrete subsets of its target mRNAs and influences their posttranscriptional fate (Yoon et al., 2013). The recent research by Chen et al. observed a significantly enrichment of β‐catenin mRNAs in HuR immunoprecipitates, indicating that HuR can bind to β‐catenin mRNA in BMSCs (Chen et al., 2022). Our previous study predicted β‐catenin as an important target mRNA of HuR (Huai et al., 2022). To test the conjectures, we analyzed and observed a significant positive correlation between HuR and β‐catenin expression in bone specimens from postmenopausal women (Figure S2F). Moreover, overexpression of HuR significantly increased both mRNA and protein levels of β‐catenin, while silencing of HuR by specific siRNA suppressed the expression of β‐catenin (Figure 3a,b), suggesting that HuR positively regulates β‐catenin expression. In addition, HuR levels exerted a positive regulatory effect on the mRNA stability of β‐catenin under varying durations (0, 6, 12, and 24 h) of exposure to ActD (a well‐established inhibitor of mRNA transcription) (Figure 3c). In osteoblasts with HuR knockdown, there was a notable decrease in the nuclear localization of β‐catenin compared to the control osteoblast group, indicating a significant disruption in the subcellular distribution of β‐catenin. To investigate the mechanism by which HuR regulates the intracellular distribution of β‐catenin, the co‐immunoprecipitation (Co‐IP) experiment was performed to examine protein–protein interactions of HuR and β‐catenin. The first observation is a significant increase in the co‐precipitation of HuR with β‐catenin in SA‐stimulated osteoblasts compared to the control group, suggesting that HuR and β‐catenin interact more extensively under conditions of SA‐induced SGs formation (Figure 3d). Furthermore, we made the additional observation that in osteoblasts with low HuR expression exposed to SA stimulation, there was a significant reduction in the co‐precipitation of HuR with β‐catenin as well as G3BP1 (Figure 3e). More importantly, there was a reduction in the co‐localization of β‐catenin (green fluorescence) and HuR (red fluorescence) within HuR‐positive SGs in HuR‐knockdown osteoblasts compared to control cells (Figure 3f). These findings further emphasize the roles of HuR in facilitating the recruitment and localization of β‐catenin within SGs, highlighting the importance of HuR in regulating SGs formation and function in osteoblasts under SA‐induced stress conditions.

### 3.4 HuR levels and osteogenesis are enhanced by API treatment during skeletal aging

Our findings suggest that targeting HuR and SGs may represent a promising therapeutic approach for mitigating decreased osteogenesis. As such, we aimed to identify a natural product capable of modulating HuR activity to enhance bone formation. A broad‐spectrum screening using the systems pharmacology approach was conducted to identify natural products with dual properties of anti‐osteoporosis and HuR activation (Figure S3A). Four potential HuR activators were initially identified among these natural products using various systems pharmacology‐related platforms such as TCMSP, TCMID, CTD, and Binding DB databases. In‐silico ADME analysis was then conducted to investigate the pharmacokinetic properties of these natural products, and API was found to exhibit a higher drug‐likeness weight (score = 0.74) compared to other natural products (Figure S3B). Furthermore, we investigated the botanical origins of these compounds and discovered that both API and epigallocatechin gallate are derived from multiple medicinal food homologous plants.

In addition, molecular docking simulations were performed to investigate the interactions between these natural products and HuR protein. The 3D‐binding conformations and 2D structures of the four natural products and their interactions with HuR were illustrated in Figures S3C and S4, respectively. All four natural products exhibited a preference for binding to the pocket located within the entrance cavity of HuR, where they established interactions with various residues. The binding energies predicted by molecular docking revealed that API showed stronger targeting action (bing energy = −6.32 kCal/mol) to HuR compared to other compounds (Figure S3B).

**FIGURE 4:**
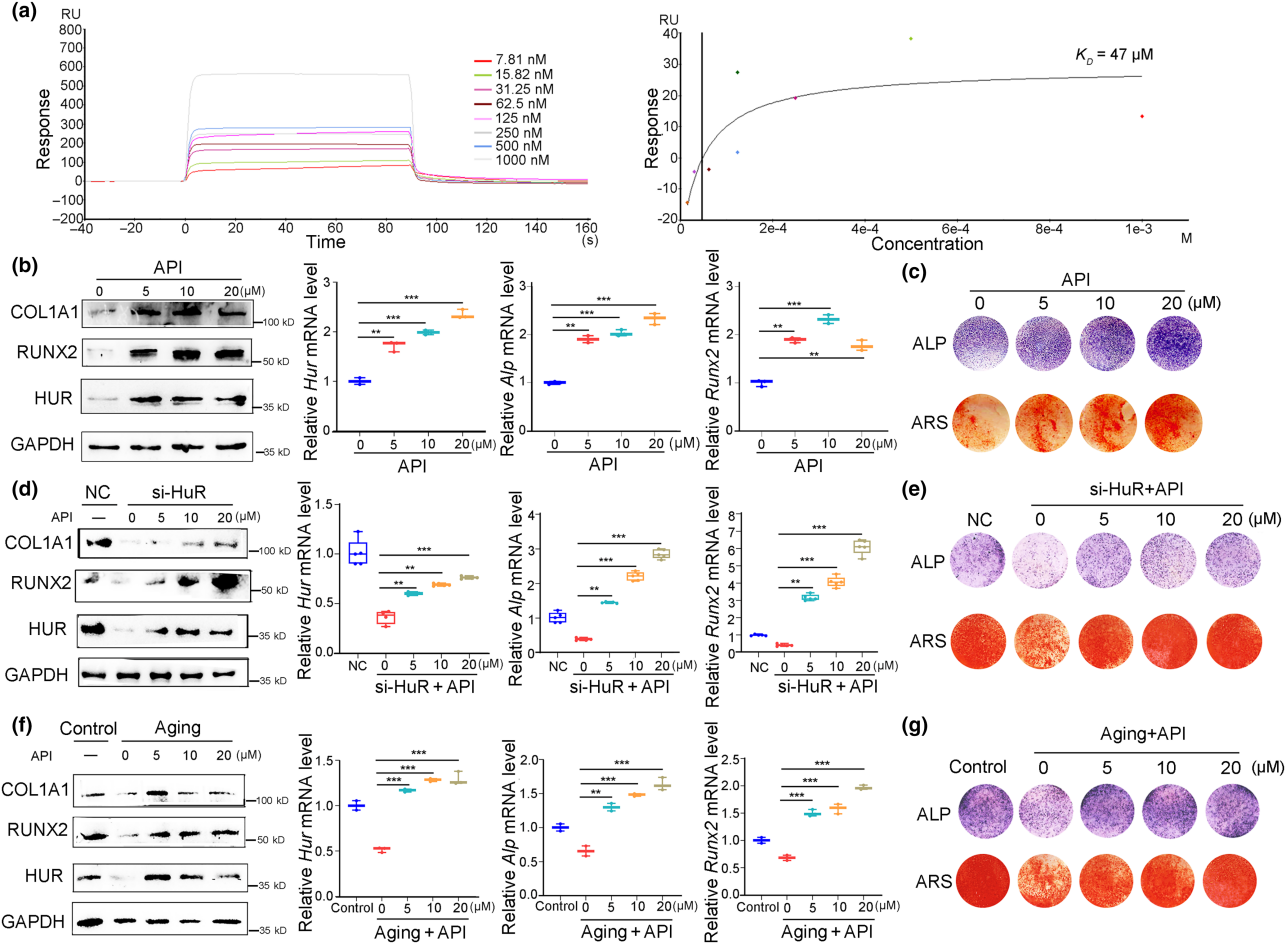
HuR levels and osteogenesis are enhanced by API treatment during skeletal aging. (a) Binding curves (colored lines) obtained by passing different concentrations of API over HuR protein immobilized on a biosensor surface (left); Standard curve and *K*_*D*_ values of the interaction between API and HuR (right). (b) Real‐time PCR and western blot analyses of HuR and osteogenic biomarkers (*Alp*, *Runx2*, and *Col1α1*) protein levels (left) and mRNA levels (right) in MC3T3‐E1 cells treated with API (0, 5, 10 and 20 μM) for 48 h *n* = 3 for each group. (c) Representative images of ALP staining (upper) and ARS staining (below) of MC3T3‐E1 cells treated with API (0, 5, 10, and 20 μM). *n* = 3 for each group. Scale bar: 8 mm. (d) Real‐time PCR and western blot analyses of HuR and osteogenic biomarkers (*Alp*, *Runx2*, and *Col1α1*) protein levels (left) and mRNA levels (right) in HuR‐knockdown MC3T3‐E1 cells after treated with API (0, 5, 10, and 20 μM) for 48 h *n* = 3 for each group. (e) Representative images of ALP staining (upper) and ARS staining (below) of HuR‐knockdown MC3T3‐E1 cells after treated with API (0, 5, 10, and 20 μM) for 48 h *n* = 3 for each group. Scale bar, 8 mm. (f) Real‐time PCR and western blot analyses of HuR and osteogenic biomarkers (*Alp*, *Runx2*, and *Col1α1*) protein levels (left) and mRNA levels (right) in etoposide‐induced senescent MC3T3‐E1 cells after treated with API (0, 5, 10, and 20 μM) for 48 h *n* = 3 for each group. (g) Representative images of ALP staining (upper) and ARS staining (below) of etoposide‐induced senescent MC3T3‐E1 cells after treated with the indicated concentrations of API (0, 5, 10, and 20 μM) for 48 h *n* = 3 for each group. Scale bar, 8 mm. GAPDH was used as the internal control for mRNA and protein. Data are represented as mean ± SD. Statistical differences among three groups were analyzed via one‐way ANOVA and significances were determined using Student's *t* test between two groups. *p < 0.05 was considered significant in all cases (**p < 0.01, ***p < 0.001).

To further investigate their anti‐osteoporotic activity, correlation coefficients between these natural products and osteoporosis were computed, and their targets related to osteoporosis were predicted. Among them, API exhibited the highest correlation coefficient with osteoporosis (correlation score = 13.68) as well as the most targets associated with osteoporosis (*n* = 767) (Figure S3B). Furthermore, the effects of these four natural products on osteogenesis and HuR expression were evaluated using ALP staining and real‐time PCR. The results revealed that API significantly enhanced ALP activity and HuR levels in MC3T3‐E1 cells compared to other compounds (Figure S3D). Based on the above analyses, API was initially identified as a HuR activator with anti‐osteoporotic activity. Subsequently, the binding affinity of API to HuR was assessed using SPR, and a potent interaction was observed, as indicated by the calculated *K*_*D*_ values (*K*_*D*_ = 47.3 μM). Additionally, their binding was found to be dose‐dependent and characterized by rapid association but relatively slow dissociation (Figure 4a). Collectively, these results establish API as a HuR activator that enhances the HuR expression and ALP activity in osteoblasts.

To further investigate the impact of API on osteoblasts activity and HuR function, we observed a dose‐dependent increase in both mRNA and protein levels of HuR as well as key osteogenic markers (ALP, RUNX2, COL1A1) following API treatment in vitro (Figure 4b). Additionally, API treatment dose‐dependently enhanced ALP activity and mineralized nodule formation in osteoblasts (Figure 4c). The effect of API treatment on the osteogenic capacity of HuR‐knockdown MC3T3‐E1 cells was analyzed. It was found that API significantly restored reduced HuR levels and recovered the expression of osteogenic markers, ALP activity, and mineralized matrix formation in a dose‐dependent manner (Figure 4d,e). Moreover, treatment with API significantly alleviated the reductions in HuR and osteogenic markers (ALP, RUNX2, and COLIΑ1) levels that were observed in senescent MC3T3‐E1 cells (Figure 4f). API treatment also dose‐dependently improved ALP activity and mineralized matrix formation in senescent osteoblasts (Figure 4g), while inhibiting the expression of aging marker genes (*P21* and *P16*) and β‐galactosidase activity in senescent MC3T3‐E1 cells (Figure S5C,D).

Together, these results demonstrate that API treatment upregulates HuR levels and thereby enhances the osteogenic potential of MC3T3‐E1 cells, suggesting that API may represent a promising therapeutic approach for mitigating decreased osteogenesis by targeting HuR and SGs.

### 3.5 Formation of HuR‐positive stress granules was promoted by API treatment

**FIGURE 5:**
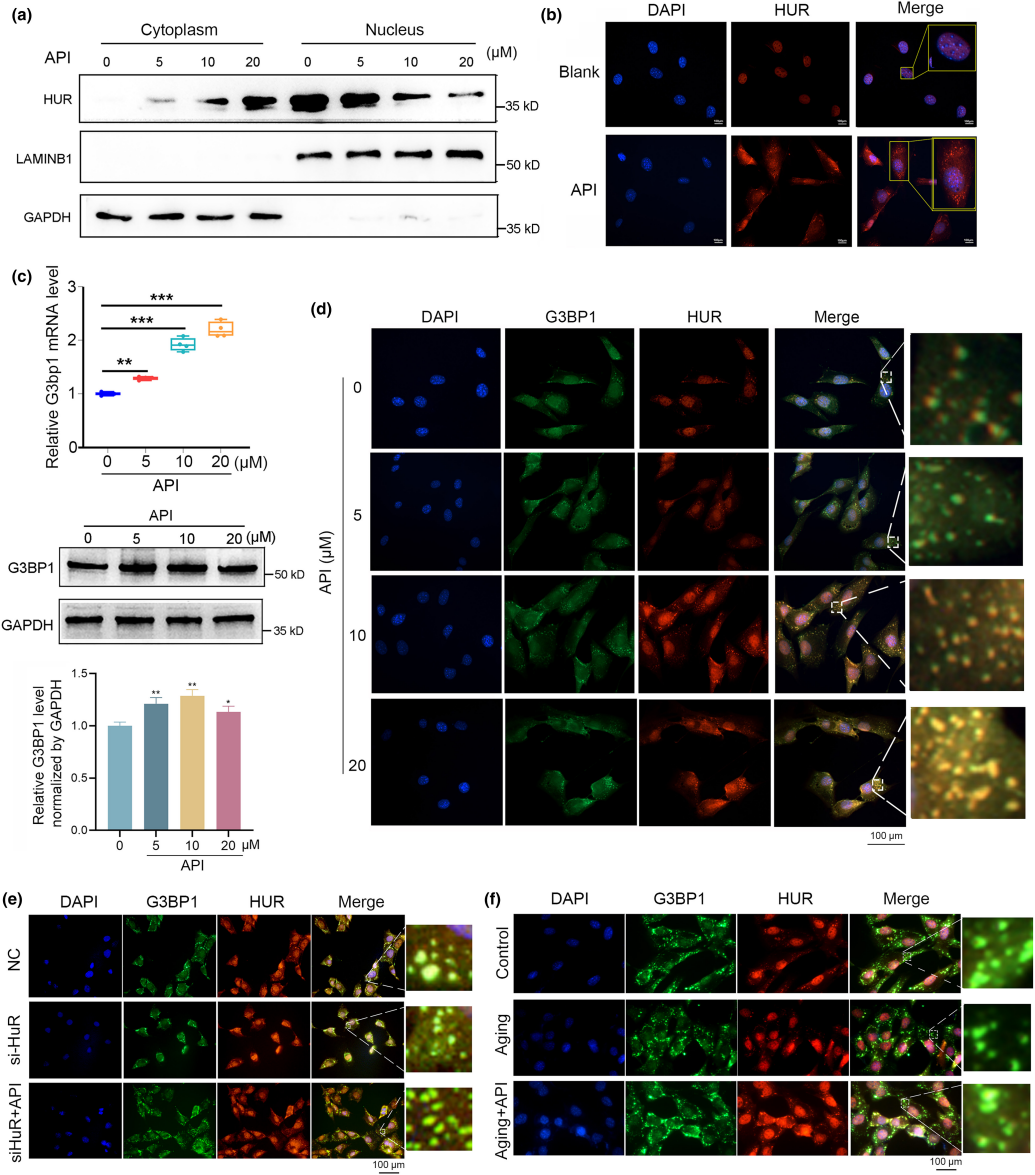
Formation of HuR‐positive stress granules was promoted by API treatment. (a) Subcellular fractionation analyses of the cellular distribution of HuR in MC3T3‐E1 cells after treated with different concentration of API (0, 5, 10 and 20 μM) for 48 h *n* = 3 for each group. (b) Cell immunofluorescence analysis of HuR localization in MC3T3‐E1 cells treating with API (20 μM) for 48 h *n* = 3. HuR: red fluorescence. Scale bar: 100 μm. (c) Real‐time PCR and western blot analyses of G3BP1 mRNA levels (Upper) and protein levels (Below) in MC3T3‐E1 cells treated with different concentration of API (0, 5, 10 and 20 μM) for 48 h and exposed to 500 μM SA for 30 min *n* = 3. (d) Cell immunofluorescence analysis of HuR‐positive SGs formation and the co‐localization of HuR and G3BP1 in MC3T3‐E1 cells treated with different concentration of API (0, 5, 10 and 20 μM) for 48 h and exposed to 500 μM SA for 30 min. G3BP1: green fluorescence; HuR: red fluorescence. *n* = 3. Scale bar: 100 μm. (e) Cell immunofluorescence analysis of HuR‐positive SGs formation and the co‐localization of HuR and G3BP1 in HuR‐knockdown MC3T3‐E1 cells (si‐HuR) after treated with API (20 μM) for 48 h and exposed to 500 μM SA for 30 min. (f) Cell immunofluorescence analysis of HuR‐positive SGs formation and the co‐localization of HuR and G3BP1 in 3‐month and 20‐month mice primary osteoblasts after treated with API (20 μM) for 48 h and exposed to 500 μM SA for 30 min. GAPDH was used as the internal control for mRNA and protein. Data are represented as mean ± SD. Statistical differences among three groups were analyzed via one‐way ANOVA and significances were determined using Student's *t* test between two groups. *p < 0.05 was considered significant in all cases (**p < 0.01, ***p < 0.001).

The findings presented above have prompted us to elucidate the underlying mechanisms by which API modulates HuR activity and osteogenic capacity in osteoblasts. Given that HuR is a RBP with nucleoplasmic shuttling property, we examined the impact of API on the cytoplasmic and nuclear expression of HuR. Subcellular fractionation analysis showed that API gradually increased cytoplasmic HuR levels while decreasing nuclear HuR levels in osteoblasts (Figure 5a and Figure S5A). Furthermore, immunofluorescence staining with HuR antibodies confirmed that API treatment promoted the translocation of HuR from the nucleus to the cytoplasm in osteoblasts, suggesting that API facilitated nuclear export of HuR (Figure 5b).

We further investigated the regulation of API on HuR‐positive SG formation in the cytoplasm of osteoblasts. Firstly, we found that API treatment enhanced the G3BP1 expression both at transcription and translation levels in osteoblasts (Figure 5c). Additionally, our observations indicated a significant increased co‐localization of HuR (red fluorescence) and G3BP1 (green fluorescence) within cytoplasmic SGs following API treatment, suggesting that API effectively mobilized dose‐dependent formation of HuR‐positive SGs in MC3T3‐E1 cells (Figure 5d). More importantly, API facilitated the co‐localization of HuR (green fluorescence) and TIA1 (red fluorescence, another important SGs biomarker) in osteoblasts (Figure S5B). We also found that API treatment alleviated the decrease of HuR‐positive SGs formation in HuR‐knockdown MC3T3‐E1 cells (Figure 5e) and rescued the reduction of HuR‐positive SGs formation in senescent MC3T3‐E1 cells (Figure 5f). Taken together, we conclude that API modulates the cytoplasmic and nuclear expression of HuR, motivates the formation of HuR‐positive SGs in osteoblasts, thereby accelerating the osteogenic process.

### 3.6 Recruitment of β‐catenin to HuR‐positive SGs was facilitated by API

**FIGURE 6:**
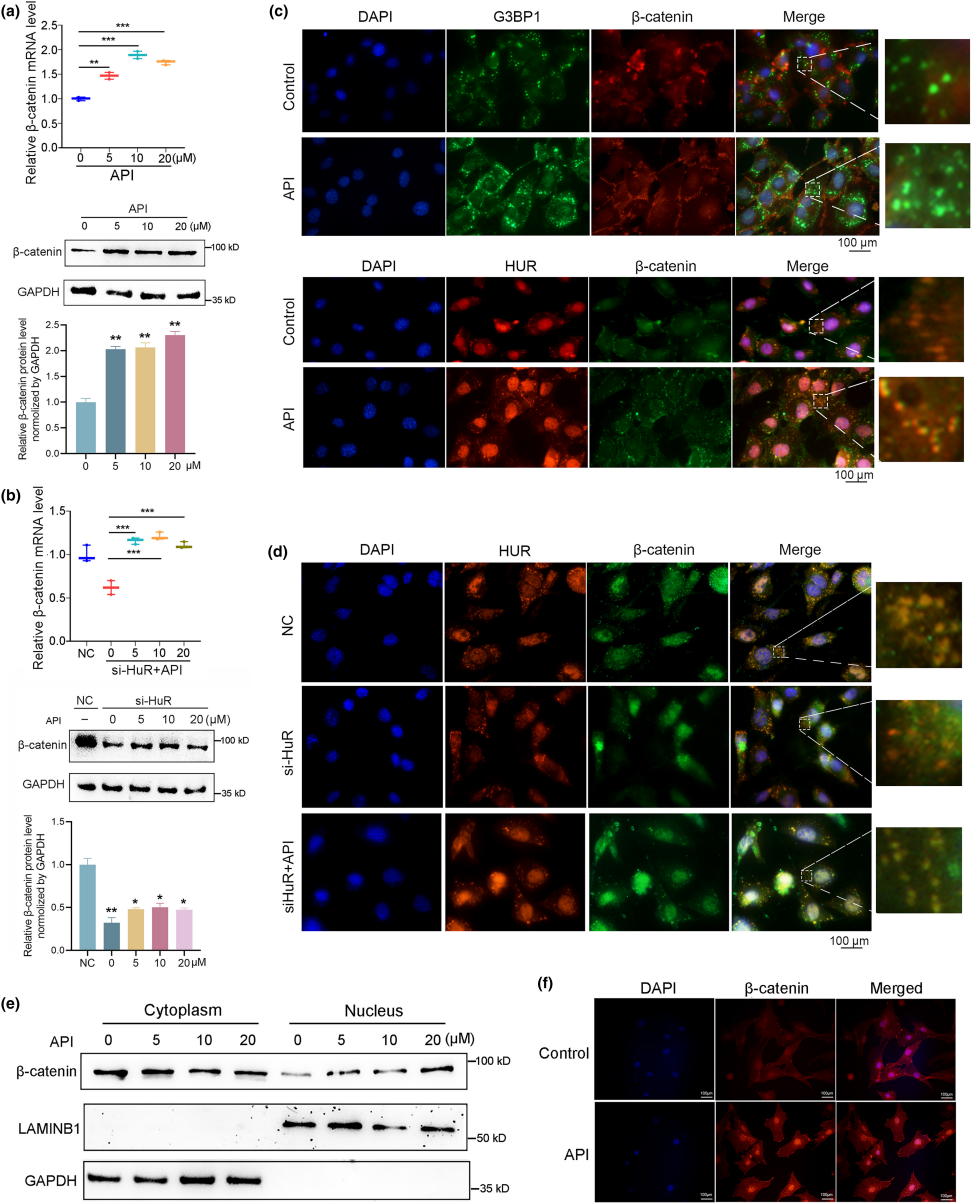
Recruitment of β‐catenin to HuR‐positive SGs was facilitated by API. (a) Real‐time PCR and western blot analyses of β‐catenin mRNA levels (left) and protein levels (right) in MC3T3‐E1 cells treated with different concentration of API (0, 5, 10, and 20 μM) for 48 h *n* = 3. (b) Real‐time PCR and western blot analyses of β‐catenin mRNA levels (left) and protein levels (right) in HuR‐knockdown MC3T3‐E1 cells treated with different concentration of API (0, 5, 10, and 20 μM) for 48 h *n* = 3. (c) Cell immunofluorescence analysis of the co‐localization of HuR, G3BP1 and β‐catenin in SGs in MC3T3‐E1 cells treated with API (20 μM) for 48 h and exposed to 500 μM SA for 30 min. Co‐localization of HuR (red fluorescence) and β‐catenin (green fluorescence) (upper); Co‐localization of G3BP1 (green fluorescence) and β‐catenin (red fluorescence) (Below). *n* = 3 for each group. Scale bar: 100 μm. (d) Cell immunofluorescence analysis of the co‐localization of HuR and β‐catenin in SGs in HuR‐knockdown MC3T3‐E1 cells and cells treated with API (20 μM) for 48 h followed by exposure to 500 μM SA for 30 min. *n* = 3. Scale bar: 100 μm. (e) Subcellular fractionation analyses of the cellular distribution of β‐catenin in MC3T3‐E1 cells after treated with different concentration of API (0, 5, 10, and 20 μM) for 48 h *n* = 3. (f) Cell immunofluorescence analysis of β‐catenin localization in MC3T3‐E1 cells treating with API (20 μM) for 48 h *n* = 3. β‐catenin: red fluorescence. Scale bar: 100 μm. GAPDH was used as the internal control for mRNA and protein. Data are represented as mean ± SD. Statistical differences among three groups were analyzed via one‐way ANOVA and significances were determined using Student's *t* test between two groups. *p < 0.05 was considered significant in all cases (**p < 0.01, ***p < 0.001).

The aforementioned results revealed that API can modulate the expression, localization, and SGs formation of HuR in osteoblasts, thereby promoting osteogenic differentiation. To deepen our understanding of the role of API on the downstream targets of HuR, we further investigate the regulatory effects of API on β‐catenin. Firstly, we observed that API treatment resulted in increased mRNA and protein levels of β‐catenin in MC3T3‐E1 cells, indicating that API positively regulates β‐catenin expression (Figure 6a). Notably, API treatment partially rescued the reduced expression of β‐catenin in HuR‐knockdown MC3T3‐E1 cells, suggesting that API may enhance β‐catenin expression through its regulatory effects on HuR (Figure 6b). Furthermore, we explored the recruitment of β‐catenin by HuR to SGs following API treatment and observed the pairwise co‐localization of HuR, G3BP1 (a SG marker), and β‐catenin within SGs. Our findings demonstrated that API significantly facilitated the mobilization of β‐catenin into G3BP1‐positive SGs (Figure 6c Upper) as well as its recruitment to HuR‐positive SGs (Figure 6c Below). Additionally, API also partially restored the decreased co‐localization of HuR and β‐catenin in HuR‐knockdown osteoblasts (Figure 6d), indicating that API enhances the ability of HuR to recruit β‐catenin into SGs.

As widely recognized, accumulation of β‐catenin in the cytoplasm and its translocation into the nucleus are pivotal steps during osteogenic differentiation. Having established the facilitation of β‐catenin recruitment by HuR into cytoplasmic SGs through API treatment, it remains to be investigated whether API exerts additional effects in promoting the subsequent nuclear translocation of β‐catenin. Our preliminary observations on the impact of API on the subcellular localization and nuclear translocation of β‐catenin revealed that API elicited a significant increase in the nuclear expression of β‐catenin while leaving its cytoplasmic levels with slightly reduction (Figure 6e). Furthermore, immunofluorescent staining showed that API facilitated the translocation of β‐catenin into the nucleus (red fluorescence) in MC3T3‐E1 cells (Figure 6f). Based on these findings, we concluded that API facilitated the recruitment of β‐catenin to HuR‐positive SGs, defending the mRNA stability and expression of β‐catenin and enhancing its nuclear translocation and subsequent osteogenesis. In summary, our results suggest that API modulates the activity of HuR and its target β‐catenin, contributing to the enhancement of osteogenic capacity observed following API treatment.

### 3.7 HuR expression and bone formation in OVX mice were improved by API

**FIGURE 7:**
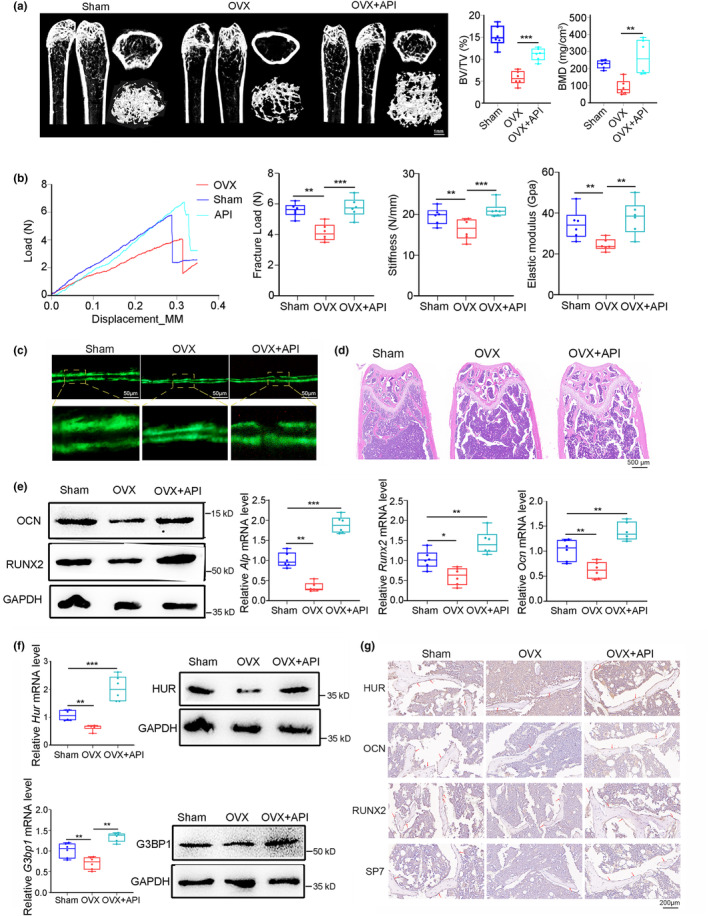
HuR expression and bone formation in OVX mice were improved by API. (a) Representative 3D reconstruction images showing microarchitecture (left) and the microCT statistical analysis of BV/TV and BMD (right) in distal femur of OVX mice after API treatment. BMD, bone mineral density; BV/TV, bone volume to tissue volume. *n* = 6 for each group. Scale bar: 1 mm. (b) Representative load‐deflection curves (left) and the mechanical strength indices (maximum force, stiffness, and maximum strength) (right) for the respective groups in tibias of Sham mice, OVX mice and OVX mice treated with API. *n* = 6 for each group. (c) Representative calcein double labeling images showing bone formation capacity in femur trabecular bone of OVX mice after API treatment. Upper scale bar, 50 μm. Below scale bar, 25 μm. (d) Representative images of H&E staining analysis in distal femur of OVX mice after API treatment. Scale bar, 500 μm. (e) Western blot and real‐time PCR analyses of osteogenic marker genes (ALP, RUNX2, and OCN) protein levels (left) and mRNA levels (right) in tibia samples from Sham group, OVX group, and OVX mice with API treatment. *n* = 6 for each group. (f) Real‐time PCR and western blot analyses of HUR and G3BP1 mRNA levels and protein levels in tibia samples from Sham group, OVX group and OVX mice with API treatment. *n* = 6. (g) Representative images of immunohistochemical (IHC) staining of HUR, OCN, RUNX2, and SP7 in distal femur of aged mice after API treatment. Scale bar, 200 μm. *n* = 3 for each group. GAPDH was used as the internal control for mRNA and protein. Data are represented as mean ± SD. Statistical differences among three groups were analyzed via one‐way ANOVA and significances were determined using Student's *t* test between two groups. *p < 0.05 was considered significant in all cases (*p < 0.05, **p < 0.01, ***p < 0.001).

To investigate the effects of API on HuR expression and bone formation in vivo, we established an ovariectomized mice model of osteoporosis. Subsequently, mice were orally administered API in CMC‐Na (sodium carboxymethyl cellulose) daily for 8 weeks via intragastric administration (Figure S6A). Serum analysis and tissue hematoxylin–eosin (H&E) staining showed no significant alterations in AST, ALT, BUN, or γ‐GT levels in mice after API treatment, indicating the absence of liver or kidney toxicity (Figure S6B,C). Then, the Micro‐CT analysis revealed that API treatment attenuated the deterioration of bone mass and trabecular bone microarchitecture induced by OVX. Moreover, API administration reversed the reduction in static bone histometric indices, including BMD, BV/TV, Tb.N, and Tb.Sp in OVX mice (Figure 7a and Figure S6D). Mechanical strength metrics, including maximum force, stiffness, and ultimate strength, were also improved following API treatment, as evidenced by three‐point bending tests (Figure 7b). Calcein double‐labeling confirmed that OVX mice treated with API showed a significantly higher bone formation rate of trabecular bone than that in OVX mice (Figure 7c). Moreover, hematoxylin–eosin (HE) staining images showed that elevated trabecular bone were observed in OVX mice after API treatment (Figure 7d). To further examine the expression of bone formation marker genes (Alp, Runx2 and Ocn) and HuR, we performed the real‐time PCR, western blot, and IHC assay analyses. We found that API treatment significantly rescued the reduced expressions of osteogenic biomarkers (Alp, Runx2, and Ocn) at both mRNA and protein levels in bone tissue of OVX mice (Figure 7e and Figure S6E). Besides, API administration led to the restoration of decreased HuR mRNA and protein levels in bone tissue, indicating its positive regulatory effect on HuR in OVX mice (Figure 7f Upper). Additionally, both G3BP1 mRNA and protein levels were significantly increased by API in bone tissue, suggesting a role for API in promoting SGs formation in vivo (Figure 7f Below). The IHC results showed that the expressions of osteogenic genes (RUNX2, OCN and SP7) and HUR in the bone tissue of OVX mice were significantly increased in the API treatment group. Collectively, our findings indicate that API treatment ameliorated HuR depletion and bone loss in OVX mice, supporting that API exerts a positive impact on bone formation and holds potential as a therapeutic agent for osteoporosis.

## 4 DISCUSSION

**FIGURE 8:**
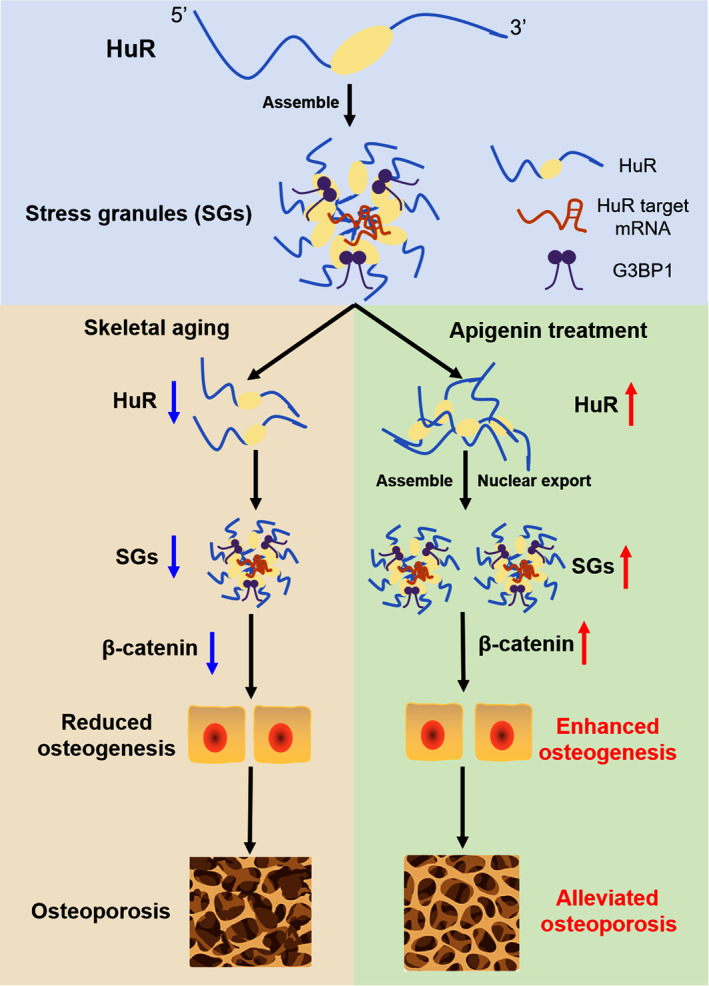
Proposed model for the roles of HuR and SG formation in osteogenesis during skeletal aging. HuR functions as a crucial bone‐related RBP that tends to as aggregate into SGs, thus regulating osteogenesis during aging. The nuclear export of HuR and SG formation is facilitated by API treatment, which enhances the recruitment of HuR to β‐catenin within SGs, ultimately promoting the β‐catenin levels and nuclear translocation in osteoblasts, and eventually enhancing bone anabolic action (see text for details).

Skeletal aging is a growing public health concern worldwide, contributing to the age‐related bone loss (Hofbauer et al., 2023). Therefore, it is imperative to uncover the underlying mechanisms and develop innovative therapeutic strategies to address this issue. In this study, we identified the unique roles of HuR in promoting osteogenesis and bone formation during skeletal aging. We observed a positive association between HuR‐positive SGs formation and osteogenesis and found that HuR‐positive SGs formation positively regulated osteoblasts differentiation. Overexpression of HuR rescued the decline in osteogenic capacity and SG formation in aging osteoblasts, highlighting the potential of HuR‐positive SGs as a promising therapeutic target for age‐related osteoporosis. Furthermore, we screened and identified API as an activator of HuR and found that API treatment significantly promoted both HuR expression and bone formation. Mechanistically, API treatment facilitated HuR cytoplasmic translocation and SG formation in osteoblasts, which enhanced the recruitment of β‐catenin into SGs by HuR, promoting an increase in β‐catenin levels and nuclear translocation, ultimately improving bone anabolic action during aging. These findings highlight the crucial roles of HuR and its SGs in regulating osteoblast function and their potential as a therapeutic target for age‐related bone loss (Figure 8).

Recent studies have highlighted the crucial role of numerous RBPs in the pathogenesis of osteoporosis, including their regulation on bone formation and bone resorption. Tang et al., revealed that RBP hnRNPK was involved in the osteogenic differentiation of bone marrow‐derived mesenchymal stem cells (BMSCs) by promoting the expression of lncRNA‐OG (Tang et al., 2019). Notably, cytoplasmic hnRNPK can also interact with GSK3β to modulate the osteoclast differentiation (Fan et al., 2015). Another study identified quaking I‐5 protein (QKI‐5) as a strong inhibitor of bone resorption by decreasing conoly stimulating factor 1 receptor (CSF‐1R) and receptor activitor of nuclear factor Kappa B (RANK) expression during osteoclastogenesis (Rauwel et al., 2020). These findings provide compelling evidence that RBPs play crucial roles in the regulation of both osteoblast and osteoclast activities. HuR is RBP that plays a crucial role in mRNA stabilization and is universally expressed (Fan & Steitz, 1998). It is involved in various physiological processes, such as adipogenesis, muscle growth, metabolic regulation, and cell growth, as well as multiple pathological conditions, including cancer (Guha et al., 2021), obesity (Siang et al., 2020), and rheumatoid arthritis (Yixin Liu et al., 2019). Previous research revealed that HuR is involved in multiple skeletal related pathological processes. For example, Liu et al., demonstrated that knockdown of HuR repressed osteosarcoma cell migration and epithelial–mesenchymal transition (Liu et al., 2021). However, the contribution of HuR to osteogenesis and age‐related bone loss remains elusive. In this study, we observed a decrease in HuR levels in OVX‐induced osteoporotic mice and primary osteoblast cells from 20‐month mice (Figure 1d–g), indicating a potential role for HuR in age‐related bone loss. Moreover, we also found that HuR levels were positively associated with osteogenesis process (Figure 1h). High HuR expression enhanced osteogenic capacity of osteoblasts, while low expression significantly suppressed osteogenesis (Figure 2a–d). These results are consistent with studies demonstrating that loss of HuR can accelerate cellular senescence (Lee et al., 2018; Shao et al., 2021). Our findings suggest that HuR acts as a positive regulator of osteogenesis and a negative modulator of aging, further supporting our previous discovery of an inverse correlation between HuR and osteoporosis (Huai et al., 2022). Interestingly, other studies have demonstrated that HuR acts as a negative regulator of adipogenesis (Li et al., 2019; Siang et al., 2020), and our previous research revealed that Lnc‐PMIF disrupted the interaction between HuR and β‐actin mRNA, thereby inhibiting β‐actin expression and suppressing osteogenic cell migration (Li et al., 2021). These observations provide crucial clinical insights into the contribution of HuR to the pathological regulation of bone formation.

HuR contains RBDs, IDRs, and PLDs, which contribute to liquid–liquid phase separation (LLPS) and SG formation (Kato et al., 2012; Lin et al., 2015). HuR binds to target mRNAs in the nucleus and escorts them into the cytoplasm, where it assumes a functional form that protects them from degradation (Brennan, 2001; Wu & Brewer, 2012). HuR has emerged as a significant components of SGs (Meyerowitz et al., 2011; Yoon et al., 2013), which are a predominant subset of cytoplasmic MLOs that regulate mRNA translation and stability (Decker & Parker, 2012; Kedersha & Anderson, 2002; Marcelo et al., 2021). Our study also revealed a positive correlation between HuR levels and the biomarkers of SGs (G3BP1 and TIA1) (Figure S1D–F), indicating the involvement of HuR in SGs formation. Significantly, we observed a positive association between SG biomarkers and osteogenic biomarkers, as well as a negative relationship between stress granule biomarkers and the age of postmenopausal osteoporotic patients (Figure S1G,H), suggesting a close link between SG formation and age‐related osteoporosis. Consistently, our observations reveal a well co‐localization of HuR with G3BP1 and TIA1 in SGs (Figure S1I) within osteoblasts, as well as an increase in the formation of HuR‐positive SGs during osteogenic differentiation (Figure 1i,j), establishing for the first time the crucial role played by HuR‐positive SGs in regulating osteoblasts. Moreover, we observed a reduction in the formation of HuR‐positive SGs in primary osteoblasts derived from 20‐month‐old mice. However, this decline could be mitigated by elevated levels of HuR during the aging process (Figure 2f–i). These findings suggest that targeting HuR‐positive SGs may present a promising therapeutic strategy for addressing age‐related bone loss.

As a well‐known RBP, HuR regulates various biological processes principally through modulating the expression and mRNA stability of its target mRNAs (Brennan, 2001). In fact, quite a few studies have shown that HuR is involved in the modulation of Wnt/β‐catenin signaling and its family members thus regulating multiple physiological and pathological processes (Kim et al., 2015; Liu et al., 2014). In this study, we found a positive correlation of HuR and β‐catenin in the bone tissues of postmenopausal osteoporotic patients (Figure S2F). Expectedly, we also observed that HuR positively regulated the expression of β‐catenin both at mRNA and protein levels in vitro (Figure 3a,b), strongly supporting that HuR is a functionally important regulator of Wnt/β‐catenin signaling (Chai et al., 2020). More importantly, HuR retarded the mRNA degradation and enhanced the mRNA stability of β‐catenin in MC3T3‐E1 cells (Figure 3c), which lends support to the fact that HuR is a well‐established mRNA stabilizer protein (Schultz et al., 2020). Factually, Chen et al., discovered a significantly enrichment of β‐catenin mRNAs in HuR immunoprecipitates, indicating the ability of HuR protein to bind with β‐catenin mRNA in BMSCs (Chen et al., 2022). Co‐IP experiment in our study also demonstrated a direct interaction between HuR and β‐catenin, with a significant enhancement in their interaction upon SA stimulation (Figure 3d). However, subsequent downregulation of HuR expression led to a notable decrease in the co‐immunoprecipitation of β‐catenin with HuR (Figure 3e), indicating that HuR facilitates the recruitment of β‐catenin into SGs in osteoblasts. These findings not only support previous research indicating that HuR can regulate the downstream molecule β‐catenin, but also unveil a potential novel mechanism by which HuR facilitates osteogenesis through sequestering the downstream molecule β‐catenin within HuR‐positive SGs.

Nowadays, numerous small molecules have now been discovered to influence the formation of SGs, including natural products such as thapsigargin (Hu et al., 2017), sorbitol (Dewey et al., 2011), and paclitaxel (Szaflarski et al., 2016). We unearthed a natural product, API, that acts as an activator of HuR through systematic pharmacology and SPR validation (Figure S3). Remarkably, API significantly boosted HuR expression, cytoplasmic translocation, and subsequent formation of HuR‐positive SGs in osteoblasts, mitigating reduced osteogenesis with great efficacy (Figures 4 and 5). Additionally, API facilitated the recruitment and localization of β‐catenin into HuR‐positive SGs, providing protection for β‐catenin and promoting its nuclear translocation in osteoblasts (Figure 6). Altogether, these findings suggest that API, as a HuR activator, can expedite the cytoplasmic accumulation of HuR, fostering the formation of HuR‐positive SGs and facilitating β‐catenin nuclear translocation to ultimately enhance osteogenesis. However, as a natural compound, API exhibits a wide range of activities and modulates multiple signaling pathways involved in bone formation apart from HuR and SGs formation. For example, it has been reported to enhance osteoblast differentiation and mineralization by modulating AnxA6 and TNAP activity (Mroczek et al., 2022). Another study reported that API enhanced osteoblast differentiation and mineralization by activating the Wnt/β‐catenin signaling pathway, which is crucial for bone development and regeneration (Pan et al., 2021). The diverse mechanisms of API in promoting bone formation can be attributed to its multi‐target, multifunctional, and multi‐activity nature as a traditional Chinese medicine monomer.

In summary, our findings have identified HuR, a bone‐related RBP, as a prominent component of SGs and a positive regulator of osteogenesis in osteoblasts, emerging as a potential therapeutic target for age‐related bone loss. Furthermore, we have demonstrated that HuR activator API can mitigate reduced osteogenesis by amplifying cytoplasmic accumulation of HuR, fostering the formation of HuR‐positive SGs, and promoting β‐catenin nuclear translocation, thereby expediting osteogenesis in osteoblasts. These findings offer novel insights into the roles of HuR in bone remodeling and provide evidence for the therapeutic potential of HuR‐positive SGs in treating osteoporosis. While our study provides significant insights into the roles of HuR and SGs in bone metabolism and regeneration, there are some limitations to our findings. For instance, in this study, we have only provided preliminary insights into the role and mechanism of HuR in regulating osteogenic differentiation, without investigating the regulatory effects of HuR on osteoclast differentiation and bone resorption. More importantly, further research is needed to explore the potential for off‐target effects, and the need for additional validation in human clinical trials. Additionally, although our study provides an initial understanding of the underlying mechanisms of HuR and SGs in bone remodeling and osteogenesis, further research is necessary to unravel the potential of HuR‐positive SGs in treating osteoporosis.

## 5 CONCLUSION

In conclusion, this study has contributed to our understanding of the roles of HuR and SGs in bone homeostasis. By demonstrating the positive regulation of HuR on osteogenesis and the formation of HuR‐positive SGs in osteoblasts, this study highlights HuR and its SGs as potential therapeutic targets for age‐related bone loss. Additionally, the discovery of API as a HuR activator and its ability to promote osteogenesis through the formation of HuR‐positive SGs and β‐catenin nuclear translocation offers a promising new approach to treating osteoporosis. These findings provide a foundation for further research into the mechanisms underlying HuR and SGs in bone metabolism and offer new potential avenues for therapeutic intervention in bone‐related disorders.

## AUTHOR CONTRIBUTIONS

Designing research studies: Qian AR, Huai Y, Chen ZH, and Li Y; Conducting experiments: Huai Y, Wang X, Zhao YP, Huang Q, and Chu XH; Acquiring data: Huai Y, Chen ZH, Wang X, Mao WJ, Wang XH, and Ru K. Analyzing data: Huai Y, Chen ZH, Zhang L, and Wang X; Providing reagents: Huai Y, Chen ZH, Lin X, Mao WJ, Wang XH, and Li Y; and writing the manuscript: Huai Y, Chen ZH, and Qian AR. All authors have approved the final version to be published.

## FUNDING INFORMATION

This work was funded by the Natural Science Foundation of China (grant number 82072106, 32101055, and 32371371), China Postdoctoral Science Foundation (grant number 2020 M683573), Natural Science Foundation of Shaanxi Province (grant number 2021JQ‐128), the Key R&D Projects in Shaanxi Province (grant number 2021SF‐242), and the Fundamental Research Funds for the Central Universities (grant number D5000210746).

## CONFLICT OF INTEREST STATEMENT

None declared.

## Supporting information


Appendix S1
