# Supplementary material for: HuR‐positive stress granules: Potential targets for age‐related osteoporosis
Source: Aging Cell. 2024 Feb 20;23(3):e14053. doi: 10.1111/acel.14053 (PMC10928564; doi:10.1111/acel.14053)
Supplement: Supplementary file 1 — Appendix S1 [file ACEL-23-e14053-s001.pdf]

Supplemental figures

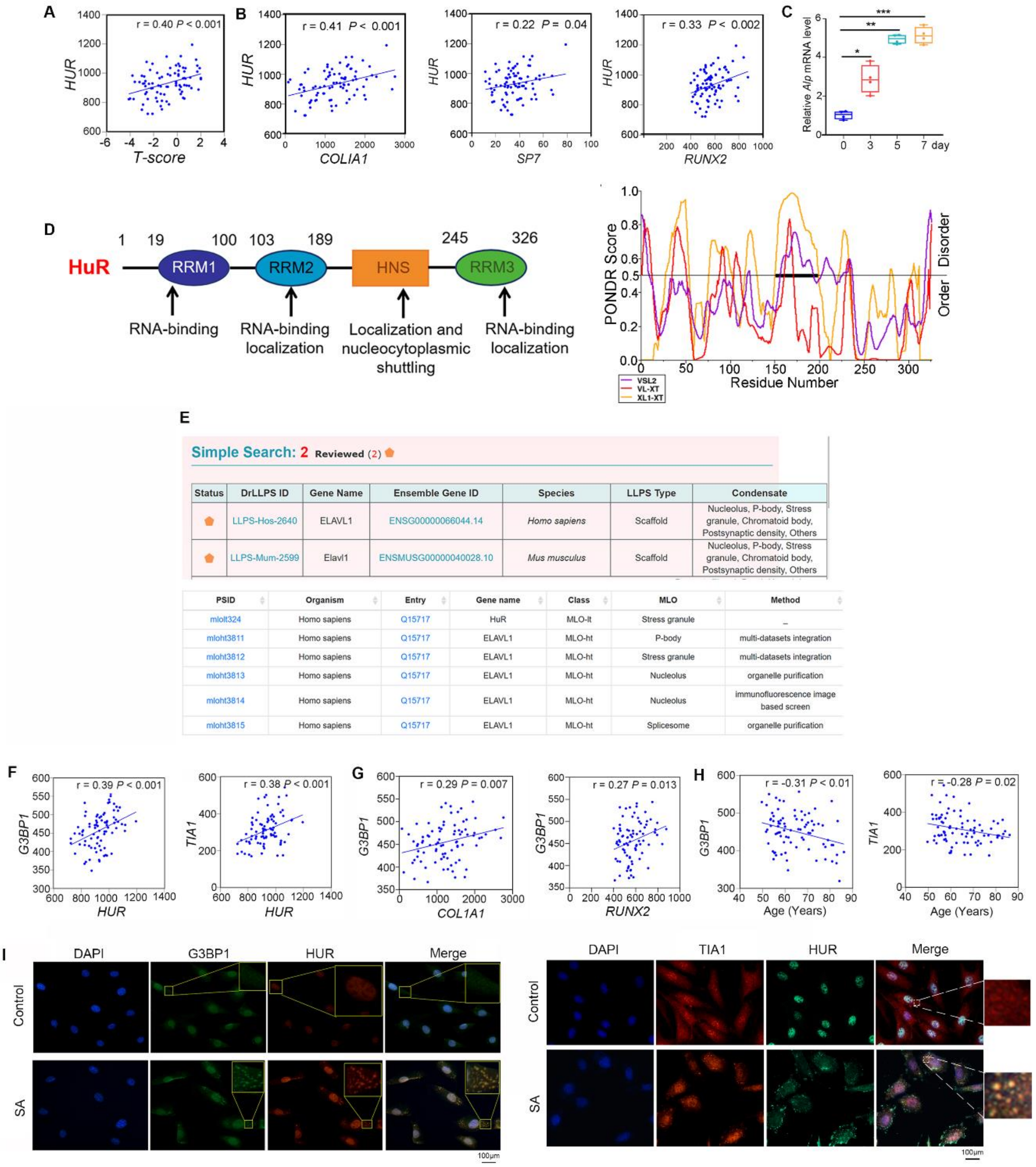

Fig.S1 HuR is positively associated with bone formation and SGs formation. A. The linear regression analyses of HuR levels and *T-score* associations in bone specimens of healthy patients or postmenopausal osteoporotic patients in dataset of E-MEXP-1618. **B.** The linear regression analyses of HuR levels and bone formation biomarkers (*COLIA1*, *SP7* and *RUNX2*) associations in bone specimens of healthy patients or postmenopausal osteoporotic patients in dataset of E-MEXP-1618.

C. Real-time PCR analysis of *Alp* mRNA levels during osteoblasts differentiation (0-7 days) of MC3T3-E1 cells. n = 3. **D.** The characteristic of HuR protein sequence with three RRMs and an HNS domain (Left). Prediction of intrinsically disordered regions (IDRs) in HuR protein sequence by PONDR server (Right). **E.** Types of membrane-less organelles (MLOs) that HuR may be involved in forming. **F.** The linear regression analyses of HuR levels and biomarker genes of SGs (G3BP1 and TIA1) association in bone specimens of healthy patients or postmenopausal osteoporotic patients in dataset of E-MEXP-1618. **G.** The linear regression analyses of G3BP1 and osteogenic biomarkers (COLIA1 and RUNX2) associations in bone specimens of healthy patients or postmenopausal osteoporotic patients in dataset of E-MEXP-1618. **H.** The linear regression analyses of biomarker genes of SGs (G3BP1 and TIA1) and age association in bone specimens of healthy patients or postmenopausal osteoporotic patients in dataset of E-MEXP-1618. **I.** Cell immunofluorescence analysis of the co-localization of HuR and biomarkers of SGs (G3BP1 and TIA1) in osteoblasts following exposure to 500μM SA for 30 min. n = 3. Data are represented as mean ± s.d. Statistical differences among three groups were analyzed via one-way ANOVA and significances were determined using student's *t*-test between two groups. $P < 0.05$ was considered significant in all cases (*$P < 0.05$, **$P < 0.01$, ***$P < 0.001$).

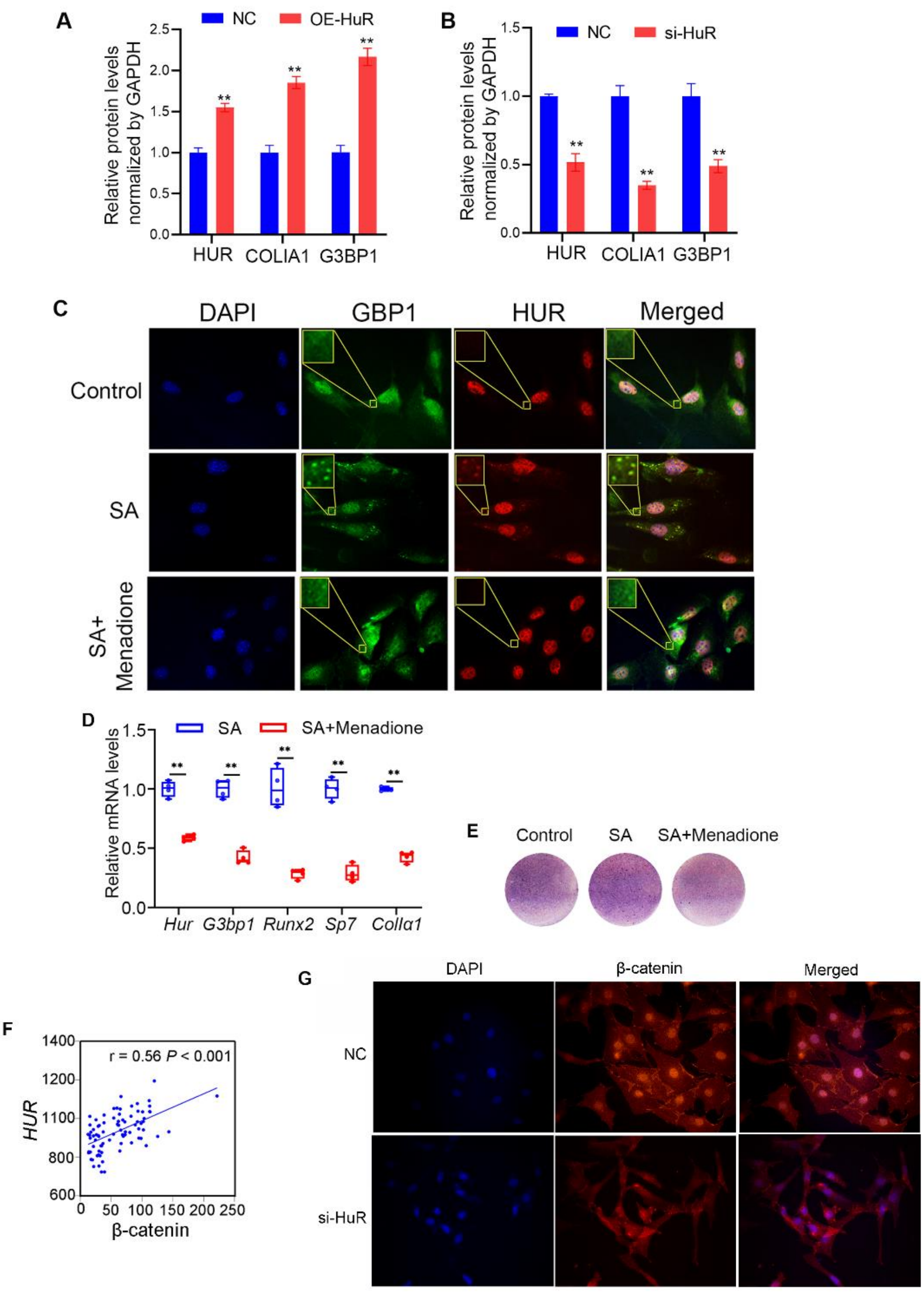

Fig.S2 HuR-positive SGs formation positively regulated osteogenesis. A-B. Quantification analysis of the western blot bands of HUR, COLIA1 and G3BP1 in HuR-overexpressed and HuR-knockdown MC3T3-E1 cells, respectively. **C.** Menadione prevents the accumulation of HuR in SGs of osteoblasts, which were treated with sodium arsenite (SA, 500 μM) with or without menadione (15 mM) for 45 min. HuR and G3BP1 were visualized by red and green immunofluorescences, respectively, and nuclei were visualized by staining with DAPI (blue). **D.** Real-time PCR analysis showed decreased levels of HuR, G3BP1 and osteogenic biomarkers (Runx2, Sp7 and Col1α1) after menadione treatment. **E.** Representative images of ALP staining of MC3T3-E1 cells treated with or

without menadione. n = 3. **F.** The linear regression analyses of *HUR* and β-catenin association in bone specimens of healthy patients or postmenopausal osteoporotic patients in dataset of E-MEXP-1618. **G.** Cell immunofluorescence analysis of β-catenin localization in MC3T3-E1 cells treating with HuR-specific siRNA. n = 3. $P < 0.05$ was considered significant in all cases (*$P < 0.05$, **$P < 0.01$, ***$P < 0.001$).

A

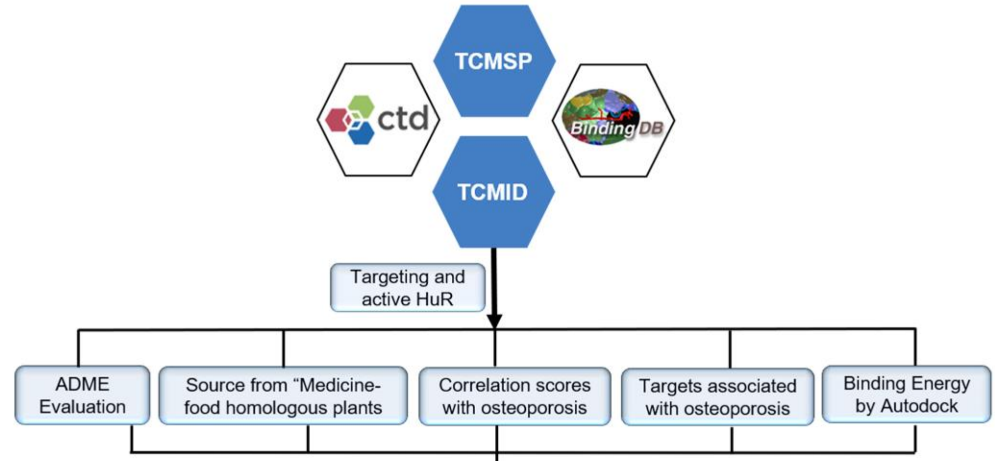

B

Compound name	ADME evaluation (Druglikeness weight)	Source from medicine-food homologous plants	Correlation score with Osteoporosis	Number of osteoporosis targets	Binding energy with HuR (kCal/mol)
Apigenin	0.740	Yes	13.68	767	-6.32
Epigallocatechin gallate	0.514	Yes	5.68	79	-6.27
Resveratrol	0.692	No	13.93	725	-6.25
Acetovanillone	0.674	No	13.61	39	-6.15

C

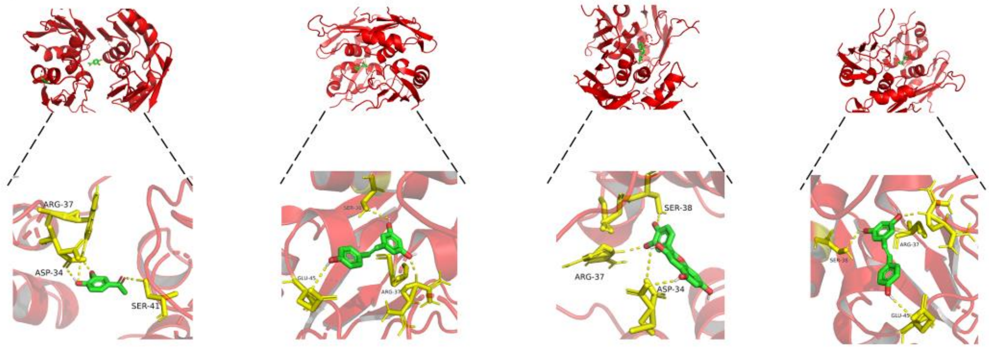

D

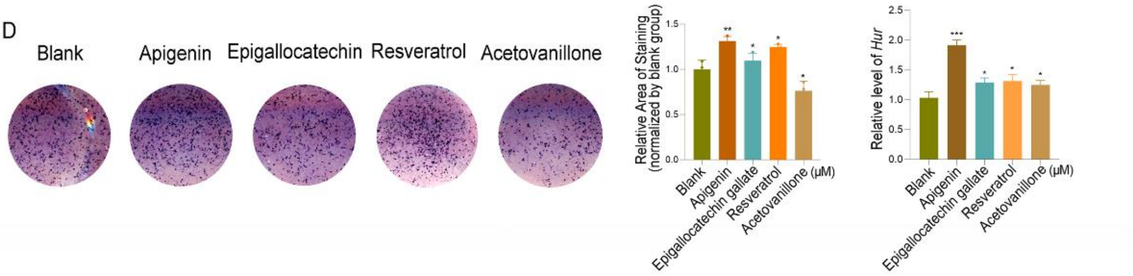

Fig. S3 Screening and identification of HuR activators. A. Screening procedure and criteria of natural products targeting HuR based on the systematic pharmacology. **B.** Detail information of four candidate compounds (apigenin, epigallocatechin-3-gallate, resveratrol, acetovanillone) targeting and activating HuR. **C.** Binding mode of four candidate compounds and HuR from molecular

simulation docking analysis by Autodock 4.2. HuR Protein structures were obtained from the Protein Data Bank (www.rcsb.org). Structures of small molecules were obtained from Pubchem database (https://pubchem.ncbi.nlm.nih.gov/). **D.** Representative ALP staining showed that API treatment showed the highest ALP activity. Real-time PCR showed the HuR expression after natural compounds treating. n = 3. Data are represented as mean ± s.d. Statistical differences among three groups were analyzed via one-way ANOVA and significances were determined using student's *t*-test between two groups. *P* value less than 0.05 was considered significant in all cases (*$P < 0.05$, **$P < 0.01$, ***$P < 0.001$).

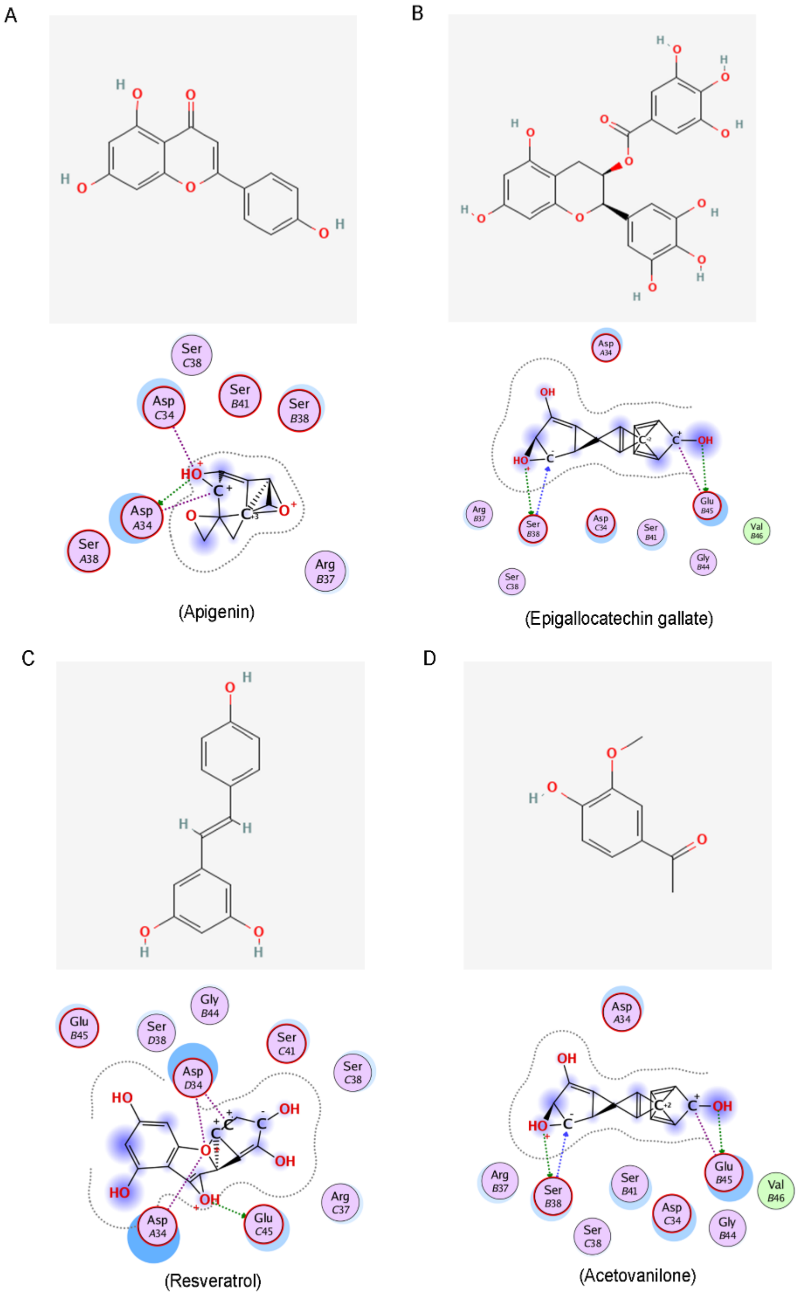

Fig. S4 Structures and 2D interaction of four natural products and HuR.

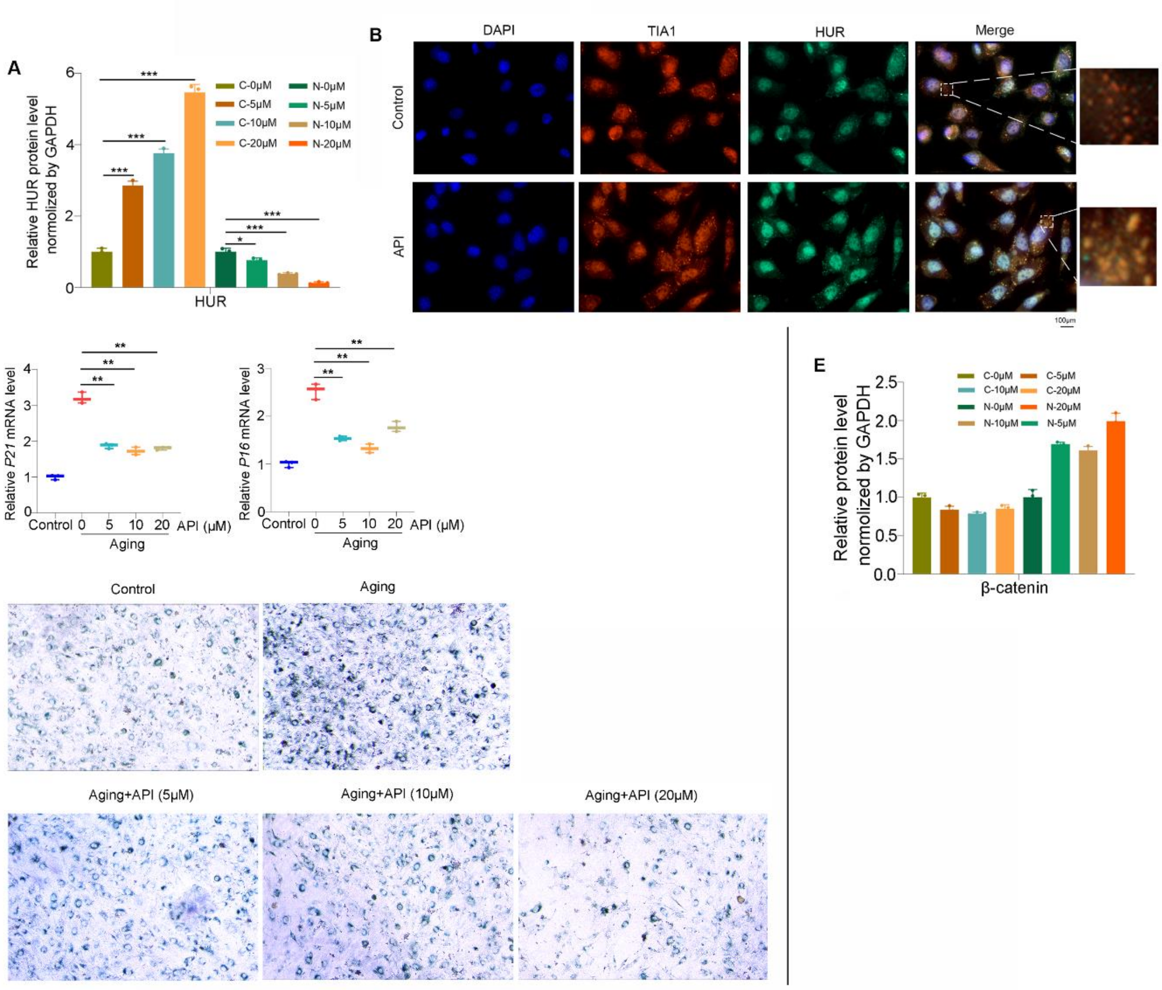

Fig. S5 API promoted cytosolic localization of HuR and alleviated the aging of senescence osteoblasts. A. The densitometric analysis of immunoblot bands of nuclear and cytoplasmic protein levels of HUR in osteoblasts after API treatment. **B.** Cell immunofluorescence analysis of HuR-positive SGs formation and the co-localization of HuR and TIA1 in MC3T3-E1 cells treated with API. **C.** Real-time PCR analyses of *P21* and *P16* mRNA levels in senescence osteoblasts after API treatment. **D.** β-galactosidase staining of senescence osteoblasts after API treatment. n = 3. **E.** Cell immunofluorescence analysis of HuR-positive SGs formation and the co-localization of HuR and G3BP1 in MC3T3-E1 cells treated with API. Data are represented as mean ± s.d. Statistical differences among three groups were analyzed via one-way ANOVA and significances were determined using student's *t*-test between two groups. *P* value less than 0.05 was considered significant in all cases (*$P < 0.05$, **$P < 0.01$, ***$P < 0.001$).

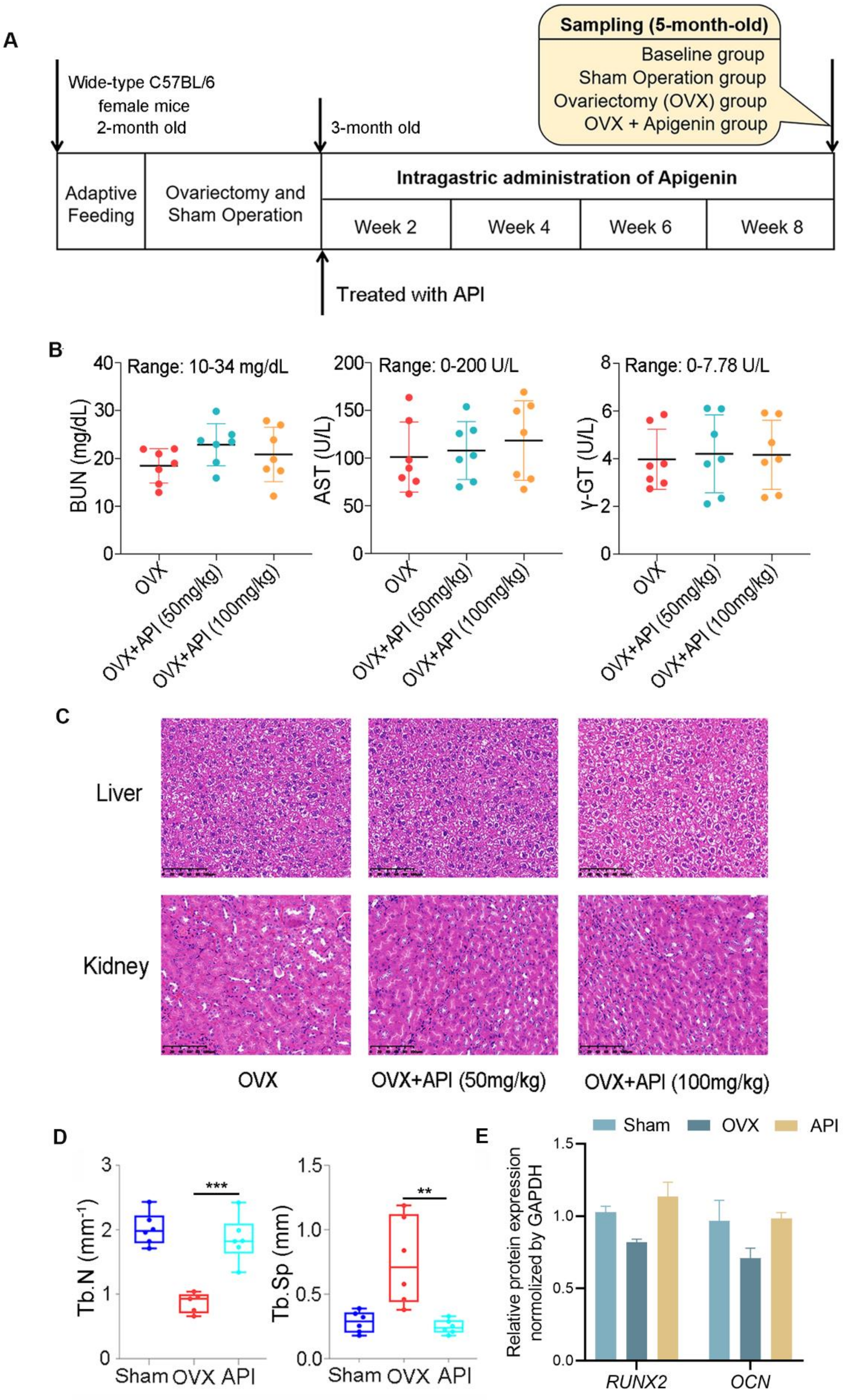
A
Sampling (5-month-old)
Baseline group
Sham Operation group
Ovariectomy (OVX) group
OVX + Apigenin group
Wide-type C57BL/6 female mice 2-month old
3-month old
Adaptive Feeding
Ovariectomy and Sham Operation
Intragastric administration of Apigenin
Week 2
Week 4
Week 6
Week 8
Treated with API
B
Range: 10-34 mg/dL
BUN (mg/dL)
Range: 0-200 U/L
AST (U/L)
Range: 0-7.78 U/L
γ-GT (U/L)
OVX
OVX+API (50mg/kg)
OVX+API (100mg/kg)
C
Liver
Kidney
OVX
OVX+API (50mg/kg)
OVX+API (100mg/kg)
D
Tb.N (mm-1)
Tb.Sp (mm)
Sham OVX API
E
Sham OVX API
Relative protein expression normolized by GAPDH
RUNX2
OCN

Fig.S6 Evaluation of the pharmacological toxicity of API on mice. A. Schematic graph showing experiment grouping and API treatment of OVX mice. Mice were intragastric administered API for 8 weeks after ovariectomy. Sham (sham-operated group), OVX group (Ovariectomy group with CMC-Na treatment), API treatment group (Ovariectomy group with API (50mg/kg) treatment for 8 weeks). **B.** Serum analysis of AST, BUN, or γ-GT content after API treatment (50 mg/kg/d and 100 mg/kg/d). **C.** Liver (Upper) and kidney (below) hematoxylin-eosin (H&E) staining after API treatment (50 mg/kg/d and 100 mg/kg/d). **D.** MicroCT statistical analysis of Tb.N and Tb.Sp in distal femur of OVX mice after API treatment. **E.** The densitometric analysis of immunoblot bands of RUNX2 and OCN in tibia samples from Sham group, OVX group and OVX mice with API treatment. n = 6 for each group. Data are represented as mean ± s.d. Statistical differences among three groups were analyzed via one-way ANOVA and significances were determined using student's *t*-test between two groups. *P* value less than 0.05 was considered significant in all cases (***P* < 0.01, ****P* < 0.001).

Supplementary for Materials and Methods

Reagents

Apigenin (API) was purchased from BaoJi Herbest Bio-Tech Co., Ltd (BaoJi, China), with high purity liquid chromate (HPLC) grade ≥ 98% pure. Sodium arsenite (SA) was purchased from Beijing Innochem Science & Technology Co., Ltd (Beijing, China). Menadione (SGs formation inhibitor) and MSAB (β-catenin inhibitor) were purchased from MedChemExpress (MCE), with high purity liquid chromate (HPLC) grade ≥ 98% pure. The following primary antibodies were used in this study: anti-HUR (1:1000, Proteintech, 11910-1-AP), anti-G3BP1 (1:1000, Proteintech, 66486-1-Ig), anti-β-catenin (1:1000, Proteintech, 51067-2-AP), anti-RUNX2 (1:1000, ABclonal, A11753), anti-COL1A1 (1:1000, ABclonal, A1352), anti-OCN (1:1000, ABclonal, A18699), anti-LAMIN B1 (1:1000, Proteintech, 12987-1-AP) and anti-GAPDH (1:1000, Proteintech, 10494-1-AP). Goat anti-rabbit and anti-mouse IgG secondary antibodies were purchased from Zhuangzhi Biotechnology (Xi'an, China). Fluorescent anti-mouse (Flux) and anti-rabbit (Cy3) secondary antibodies were purchased from ABclonal (Wuhan, China).

Cell culture and transfection

Murine pre-osteoblasts MC3T3-E1 cells were cultured in alpha-Minimum Essential Medium (α-MEM) supplemented with 2.2 g/L sodium bicarbonate, 10% fetal bovine serum (FBS), 1% penicillin and streptomycin, in a humidified, 37°C, 5% CO_2 incubator. For in vitro differentiation, 100% confluency of osteoblasts were cultured in osteogenic medium containing α-MEM, 10% FBS, 1mM dexamethasone (Sigma, D4902), 1% β-glycerophosphate (Sigma, G9422), 1% ascorbic acid (Sigma, A7631) and 1% L-glutamine, and the medium was replaced every 2 days.

Transient transfection of MC3T3-E1 cells with siRNAs (RongQingchang Biotech, Xi'an, China) or DNA plasmids of HuR (Gemma, Shanghai, China) was performed using Lipofectamine 2000 reagent (Invitrogen, USA). DNA plasmids (500 ng per well), empty plasmid (500 ng per well), siRNA control (50 nM) and siRNA (50 nM) were transfected into cells in serum-free medium. After transfection for 6 h, the serum-free medium was replaced with a growth medium and the cells were harvested for further detection.

Senescent osteoblast induced by etoposide

The osteoblasts were seeded at 24-well plates at a density of 10^5 cells/well. Cells were incubated with α-MEM medium supplied with 2 μM etoposide (Solarbio, China)

for 48 h. Then, the induced the osteoblasts were incubated with standard α-MEM medium and harvested for real-time PCR, western blot analysis or cell staining.

Cytotoxicity Assay

The cytotoxicity of API on MC3T3-E1 was determined by CCK8 assay kit (Biosharp, China). Briefly, MC3T3-E1 cells were seeded onto 96-well plates at a density of 1×10^4/well and cultured with α-MEM and treated with various concentrations of API (0, 5, 10, 20, 50, and 100 μM). After 48 h of incubation, 10μL CCK8 solution was then added to each well and incubated for 4 h at 37°C. Finally, the optical density was measured at a wavelength of 450 nm using a microplate analyser (BioTek, United States).

β-galactosidase staining: β-galactosidase staining was performed with a Senescence β-galactosidase staining Kit (Beyotime, China) according to the manufacturer's instructions. Briefly, cells were carefully rinsed with PBS three times and fixed with β-galactosidase staining stationary liquid for 15 min. The fixed cells were washed with PBS three times again. Then, the liquid substrate was added to each cell well to staining at 37°C (without CO_2) overnight. Finally, after washing with PBS three times, the cell plates with were imaged by an optical microscope (Nikon, Japan).

RNA extraction, Real-time PCR

Total RNA from bone tissues or cell samples were isolated using the Omega Total RNA Kit I (Omega, USA) following the manufacturer's instructions. RNA quality was then monitored by absorbance at 260 and 280 nm through ultraviolet spectrophotometry. The RNA was reverse-transcribed into cDNA using a cDNA synthesis kit (PrimeScript™ RT Reagent Kit, TaKaRa, Japan) following the manufacturer's instructions. The cDNA was amplified by Real-time PCR using the SYBR® Premix Ex Taq™ II kit (TaKaRa, Japan). Relative fold changes of the genes were analyzed by the $2^{-\Delta\Delta CT}$ method. *Gapdh* was used as internal controls for normalization. All primer sequences used in this work were listed in Supplementary Table 1.

Western blot analysis

For western blot analysis, cell samples of total protein were lysed on ice in Cell Lysis Buffer (Beyotime, Jiangsu, China) supplemented with 1% Protease Inhibitor Cocktail Set III (Merck, Kenilworth, Germany). Nuclear and cytoplasmic proteins were extracted using a Nuclear and Cytoplasmic Extraction Kit (Pioneer biotechnology, Shaanxi, China). Proteins were subjected to SDS-PAGE and then transferred to PVDF membranes (PALL, USA). The membranes were blocked with 5% skim milk and

incubated overnight with specific primary antibodies at 4 °C. HRP-labeled secondary antibody was added, and then, the membrane was visualized using a T5200 Multi Chemiluminescence Detection System (Tanon, China) as recommended by the manufacturer. Quantification of the protein level was performed using image analysis software (Image J, NIH, USA). The relative protein levels were normalized to the control or mock group. GAPDH and LaminB1 protein levels were used as internal controls for normalization. The relative qualification of proteins was analyzed using ImageJ software (NIH, USA).

Senile osteoporotic mice model

The wild-type male C57BL/6 mice were purchased from the Laboratory Animal Center of Air Force Medical University (Xi'an, Shaanxi, China). All the mice were maintained under standard animal housing conditions with food and water ad libitum (12 h: 12 h light: dark cycle). Natural aging mice (≥ 20 months old) were used to construct the senile osteoporotic model, and normal 3-month mice were used as control. The survival condition of the mice was monitored continuously, and any unhealthy mouse was excluded from our study. All aspects of the animal research were conducted following the guidelines set by the Laboratory Animal Ethics & Welfare Committee of Northwestern Polytechnical University.

Ovariectomy (OVX) mice model and API administration

Mice ovariectomy (OVX) was performed to construct the postmenopausal osteoporosis model. All mice that were purchased from Laboratory Animal Center of the Air Force Military Medical University (Xi'an, China) were maintained under standard housing conditions (25°C, 50-55% humidity, and a 12-h light/12-h dark cycle) and were randomized to the treatment and control group. After domesticated for one week, 8-week-old female C57BL/6J mice were anesthetized and underwent bilateral OVX or a sham operation from back approach. After a week of convalescence and incision healing, mice were given intragastric administration with BM at 15 mg/kg and 30 mg/kg respectively every day for 8 weeks. Then whole blood samples by cardiac puncture were obtained immediately after euthanasia and serum was collected and stored at -80 °C before analyses. In addition, femurs, tibias, livers and kidneys of the mice were also collected. All animal experimental protocols were complied with all relevant ethical regulations of "the Guiding Principles for the Care and Use of Laboratory Animals" (the Institutional Experimental Animal Committee of Northwestern Polytechnical University, Xi'an, China) and all experimental procedures

were approved by the Institutional Experimental Animal Committee of Northwestern Polytechnical University, Xi’an, China.
